# Internal exposure dynamics drive the Adverse Outcome Pathways of synthetic glucocorticoids in fish

**DOI:** 10.1038/srep21978

**Published:** 2016-02-26

**Authors:** Luigi Margiotta-Casaluci, Stewart F. Owen, Belinda Huerta, Sara Rodríguez-Mozaz, Subramanian Kugathas, Damià Barceló, Mariann Rand-Weaver, John P. Sumpter

**Affiliations:** 1 Brunel University London, Institute of Environment, Health and Societies, London, UB8 3PH, United Kingdom; 2 AstraZeneca, Global Environment, Alderley Park, Macclesfield, SK10 4TF, United Kingdom; 3 Catalan Institute for Water Research (ICRA), Scientific and Technological Park of the University of Girona, Girona, 17003, Spain; 4 Water and Soil Quality Research Group, Department of Environmental Chemistry, IDAEA-CSIC, Jordi Girona 18–26, 08034 Barcelona, Spain; 5 Brunel University London, College of Health and Life Sciences, London, UB8 3PH, United Kingdom

## Abstract

The Adverse Outcome Pathway (AOP) framework represents a valuable conceptual tool to systematically integrate existing toxicological knowledge from a mechanistic perspective to facilitate predictions of chemical-induced effects across species. However, its application for decision-making requires the transition from qualitative to quantitative AOP (qAOP). Here we used a fish model and the synthetic glucocorticoid beclomethasone dipropionate (BDP) to investigate the role of chemical-specific properties, pharmacokinetics, and internal exposure dynamics in the development of qAOPs. We generated a qAOP network based on drug plasma concentrations and focused on immunodepression, skin androgenisation, disruption of gluconeogenesis and reproductive performance. We showed that internal exposure dynamics and chemical-specific properties influence the development of qAOPs and their predictive power. Comparing the effects of two different glucocorticoids, we highlight how relatively similar *in vitro* hazard-based indicators can lead to different *in vivo* risk. This discrepancy can be predicted by their different uptake potential, pharmacokinetic (PK) and pharmacodynamic (PD) profiles. We recommend that the development phase of qAOPs should include the application of species-species uptake and physiologically-based PK/PD models. This integration will significantly enhance the predictive power, enabling a more accurate assessment of the risk and the reliable transferability of qAOPs across chemicals.

Mechanistic and pathway-centric approaches represent the cornerstones of 21^st^ century toxicology^1^. In recent years, the replacement of animal tests with computational models and high-throughput *in vitro* screenings has been proposed as the future of chemical toxicity testing^1^,^2^. The efficient and reliable use of *in silico* and *in vitro* approaches for risk assessment is strictly dependent on our understanding of the *in vivo* consequences of pathways perturbation across multiple levels of biological organization (mode of action, MoA). In this context, the concept of adverse outcome pathway (AOP) has been proposed as a tool to integrate existing knowledge concerning the linkage between a direct molecular initiating event (MIE) and a chain of key events at increasing levels of biological organization, from sub-cellular responses up to population dynamics^3^. The AOP concept can have immediate utility as a theoretical tool for hypothesis-driven toxicological research; however, its application to risk assessment and decision-making is currently limited by its qualitative nature^3^,^4^. The development of quantitative AOPs (qAOPs) has been advocated by several authors^4^,^5^ and, if achieved, will allow the full exploitation of the AOP predictive potential.

In their current formulation AOPs are not focused on specific chemicals, but on the identification of the molecular targets potentially modulated by those chemicals and on the consequent multi-level effects *in vivo*^6^. This implies that exposure levels, either external or internal, are not currently considered during AOP development. However, the transition from qualitative AOPs to predictive quantitative AOPs will require the integration of both pharmacodynamics (PD) and pharmacokinetics (PK), as is the combination of these two distinct but inter-dependent aspects that drive the quantitative prediction of the toxicological risk. Determining the concentration of a chemical that, if present in the organism, can lead to adverse effects allows the transition from hazard identification (*in vitro*) to risk assessment (*in vivo*) for a realistic exposure scenario (e.g. daily chemical intake). In human health risk assessment many efforts have been allocated to the *in vitro* to *in vivo* extrapolation (IVIVE) of effect concentrations established using *in vitro* assays^2^,^7^,^8^. In these cases, IVIVE is highly relevant to drive risk assessment since, in line with the precautionary principle, the risk of effects at levels of organization higher than those considered in the *in vitro* assay has very narrow limits of tolerance. Nevertheless, in other cases a certain level of risk is accepted and the attention is focused instead on risk management. In this context, the modulation of a molecular target (or pathway) does not automatically imply that the potential consequent effects will be adverse or adverse enough to be not tolerable. Examples of risk management and tolerated chemical-induced effects are, respectively, determination of drug safety margins in humans and effects in wildlife that do not affect population dynamics. AOPs are relevant for risk management since they are intended to cover all levels of biological organization, so that, once an AOP is established, risk can be assessed on the basis of the most relevant part of the pathway (e.g. molecular level *vs* population level) for different target-species (e.g. humans *vs* wildlife) and the most relevant exposure route.

Considering the critical role of PK/PD in predictive toxicology, in this study we investigated the role played by internal exposure dynamics and chemical-specific properties (i.e. physicochemical, PK and PD) in the development of qAOPs. As proof of principle, we used a fish model—fathead minnow—and a synthetic glucocorticoid receptor (GR) agonist (the pharmaceutical beclomethasone dipropionate; BDP) to develop a BDP-specific qAOPs network based on internal drug concentrations. BDP is a widely prescribed pro-drug that in the body is rapidly activated by metabolic transformation into the potent beclomethasone 17-monopropionate (17-BMP), which is the chemical form driving the therapeutic effects. 17-BMP is successively converted into free beclomethasone (BOH) and excreted (Supplementary Fig. S1). Synthetic glucocorticoids and the modulation of GR offered an ideal learning case. In fact, GR-mediated signalling plays a critical role in a number of metabolic and homeostatic functions that are necessary for life and its perturbation is involved in a number of diseases^9^. Synthetic glucocorticoids are able to modulate the GR and are widely prescribed to treat, for example, numerous chronic inflammatory conditions, such as asthma. The chronic modulation of GR, together with the ability of some synthetic glucocorticoids to interact also with other steroid receptors (i.e. androgen receptor, AR; progesterone receptor, PR) can lead to adverse effects. Our study aimed at disentangling the complexity of *in vivo* responses to chronic exposure to synthetic glucocorticoids and to synthesise this information into a predictive qAOP network. We focused on three major pathways leading to perturbation of blood glucose homeostasis, immunodepression, and skin androgenisation. We demonstrated how internal exposure dynamics and chemical-specific PK/PD properties have profound implications for the occurrence of multi-scale effects, driving their magnitude and adversity. We propose that the explicit consideration of PK and PD during the development phase will enhance the predictive power of qAOPs and their transferability across chemicals and across species.

## Results

### Water concentration of BDP and exposure dynamics

To test the role played by internal exposure dynamics in the development of qAOPs, we performed and compared two independent 21-day *in vivo* experiments during which adult fish were exposed to BDP for 21 days under different exposure dynamics. The two studies had the same peak concentrations (C_max_) of BDP but different time-integrated concentrations (AUCs). BDP water concentrations in Experiment 1 followed an oscillatory dynamic, reaching the peak concentration (10, 100, 1000 ng/L) every 4 days (Supplementary Fig. S4). Fish were exposed to the peak concentrations for 10 hours only out of the total 504 hours of exposure. In Experiment 2, water BDP concentrations (10 and 1000 ng/L) remained constant throughout the duration of the study (Supplementary Table S3, Supplementary Fig. S4). Mean measured water concentrations in the 10 and 1000 ng/L groups were, respectively, 9.1 ± 1.1 and 967 ± 22 ng/L (n=5).

### BDP uptake, metabolism and pharmacokinetic profile

**Figure 1:**
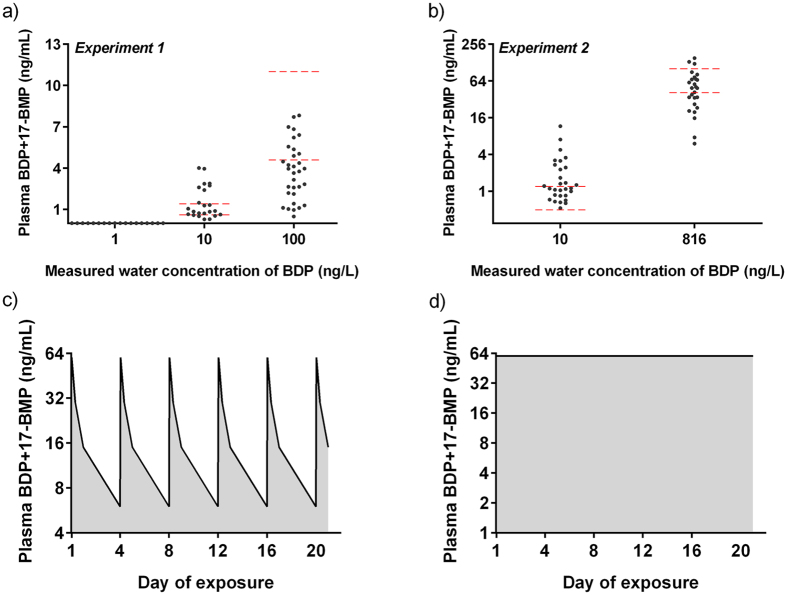
Plasma concentrations of BDP +17-BMP in fish exposed to BDP for 21 days. (**a,b**) Relationship between measured (black dots; n = 36) and predicted (dashed red lines) plasma concentrations of BDP +17-BMP in fish from Experiment 1 and Experiment 2. The range of predicted plasma concentrations was generated by using the Fish Plasma Model^10^ with Log K_ow_, Log D_7.4_ and measured water concentrations of BDP on sampling day as inputs. (**c,d**) Predicted plasma concentrations for fish exposed to 1000 ng BDP/L (nominal concentration) in Experiment 1 and Experiment 2. Fish in the two experiments were exposed to the same peak concentrations of BDP (C_max_) but to different total drug concentration over time (AUCs).

To characterize the internal exposure to BDP, we quantified the concentrations of BDP and its metabolites (17-BMP, BOH) in the plasma of individual adult fish after 21 days of exposure. Measured concentrations were compared to those predicted by the Fish Plasma Model, a steady-state uptake model^10^. Measured drug plasma concentrations were predicted with a good degree of accuracy by the concentration of BDP in water at the sampling time (Day 21), indicating lack of accumulation of the drug in the fish over time, in agreement with the fast metabolism of BDP in humans (BDP t_1/2_ = 0.5 h; 17-BMP t_1/2_ = 2.5 h)^11^. Considering the fast metabolic transformation of BDP into 17-BMP, the sum of the two was considered as the most relevant value for comparing measured and predicted concentrations and to interpret uptake. In Experiment 1 (oscillating exposure), fish were sampled when water concentrations of BDP were approximately 10% of the peak concentrations. This was reflected in the plasma concentrations of BDP +17-BMP (Fig. 1a), which were <LOD in all fish in the 10 ng BDP/L (peak concentration) exposure group, but successfully quantified in fish in both the 100 and 1000 ng BDP/L (peak concentrations) exposure groups. Average plasma concentrations of BDP + 17-BMP in the latter two groups were, respectively, 1.6 ± 1.8 ng/mL and 4.0 ± 2.0 ng/mL. BOH concentrations were <LOD in all samples; however, BOH was detected and quantified in water collected at the sampling time, suggesting fast excretion of BOH from fish into water. 17-BMP was also quantified in the same water samples, indicating partial excretion. Inter-individual variability of BDP +17-BMP was calculated for each individual tank and was between 2- and 7-fold. In Experiment 2 (sustained exposure), average plasma concentrations of BDP +17-BMP in fish from the 10 and 1000 ng BDP/L exposure groups were, respectively, 1.2 ± 0.8 ng/mL and 60 ± 35 ng/mL (Fig. 1b). BOH was <LOD in fish in the 10 ng BDP/L group, but it was quantified in 6 out of 30 fish in the 1000 BDP ng/L group (3.3 ± 3.8 ng/mL). Inter-individual variability of BDP +17-BMP was calculated for each individual tank and was between 4- and 8-fold.

In fish exposed to 10 ng/L in Experiment 2, measured partitioning coefficient water:plasma (P_water:plasma_) was 150. The measured average P_water:plasma_ in the group exposed to 1000 ng/L was 54 *versus* predicted values of 65 (based on Log K_ow_) and 124 (based on Log D_7.4_). It is plausible that the inclusion of plasma BOH in the calculations – not possible in this case because of fast excretion – would have led to an even closer agreement between predicted and measured concentrations. The comparison of measured plasma concentrations of BDP +17-BMP in fish from Experiment 1 and Experiment 2 sampled from water containing similar concentrations of BDP (approx. 10 ng/L) confirmed that drug plasma concentrations were determined by BDP water concentrations at the sampling time. On the basis of this conclusion, we used measured water concentrations of BDP over 21 days to predict drug plasma concentrations for the entire duration of the studies, allowing us to estimate the oscillating or sustained exposure dynamic in fish blood (Fig. 1c,d). The predicted internal exposure profile was used to calculate time-integrated plasma concentrations expressed as AUC values. It was predicted that in Experiment 1, fish were exposed to 0.16, 1.6 and 16 ng·h/mL, whereas in Experiment 2, AUC values were 1 and 60 ng·h/mL. Average drug plasma concentration in fish exposed to 10 ng BDP/L in Experiment 2 was within the Human Therapeutic Plasma Concentrations (H_T_PC) range expressed as C_max_ (0.8–1.4 ng/mL), whereas plasma concentrations fish exposed to 1000 ng BDP/L were approximately 50-fold higher than the H_T_PC range, suggesting the likelihood of significant toxicological effects. On the basis of this result, it is predicted that drug plasma concentration in fish exposed to 10 ng BDP/L in Experiment 1 reached the H_T_PC range once every 4 days, whereas in the same 4-day period it oscillated between 1- and 10-fold H_T_PC in the group exposed to 100 ng BDP/L and between 10-and 100-fold H_T_PC in the group exposed to 1000 ng BDP/L.

### AOP 1: effects on gluconeogenesis

**Figure 2:**
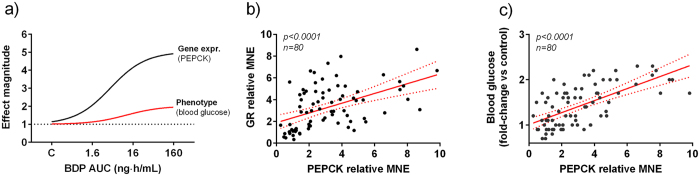
Effects of BDP on GR, PEPCK and blood glucose in fathead minnows. (**a**) Relationship between PEPCK gene expression and increase of blood glucose in fish exposed to increasing plasma concentrations of BDP. Best-fit regression curves are shown for PEPCK gene expression measured in the liver (solid black curve) and blood glucose concentration (solid red curve). Effect magnitude was calculated for each individual sample as fold-changes versus the control mean value. (**b**) Highly significant correlations were observed between PEPCK and GR gene expression and (**c**) between PEPCK gene expression and blood glucose concentrations. The solid red line represents the linear regression curve, the dashed lines the 95% confidence interval.

One of the main side effects of prolonged exposure to synthetic glucocorticoids is the disruption of glucose homeostasis *via* induction of phosphoenolpyruvate carboxykinase (PEPCK). To investigate this effect, we focused on the pathway involving GR, PEPCK and blood glucose, three inter-dependent MoA-related endpoints. Specifically, we assessed the quantitative relationship between the gene expression of GR and PEPCK and the drug-induced increase of blood glucose, with the objective of elucidating the linkage between gene expression and phenotypic responses. GR expression in the liver was significantly induced by 3.2-fold (*p* < 0.001) after oscillating exposure to 100 and 1000 ng BDP/L in Experiment 1 (Supplementary Fig. S6). The maximum observed GR expression induction was 7-fold. No significant effects were observed in fish that underwent oscillating exposure to 10 ng BDP/L. These results were confirmed in Experiment 2, in which sustained exposure to 1000, but not to 10, ng BDP/L significantly induced GR by 2.2-fold (*p* < 0.001). Liver PEPCK expression was significantly elevated in fish exposed to 1000 ng BDP/L in both oscillating (2.4-fold, *p* = 0.04) and sustained (2.7-fold, *p* = 0.002) conditions (Supplementary Fig. S6). Blood glucose was elevated after oscillating exposure to 100 ng BDP/L or higher in a concentration-dependent manner, as observed in a previous *in vivo* experiment^12^ during which fish were exposed to BDP with the same conditions used for Experiment 1. The re-analysis of the combined data-sets allowed us to generate the dose-response curves for each component of the pathway (Fig. 2a). Significant correlations were observed between PEPCK gene expression and GR gene expression (*p* < 0.001) and between PEPCK gene expression and blood glucose (*p* < 0.001) (Fig. 2b,c).

### AOP2: effects on the immune system

**Figure 3:**
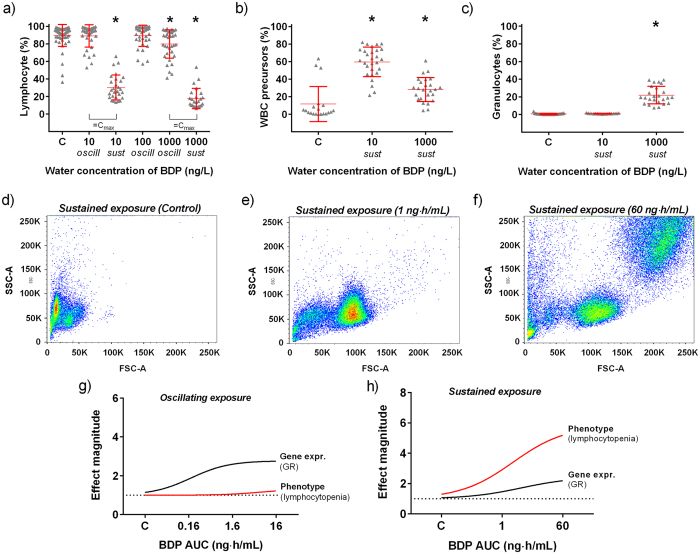
Effect of BDP on the immune system of fathead minnows. White blood cells were analysed by flow-cytometry and changes in the percentages of lymphocytes, precursors and granulocytes were quantified in each individual fish. (**a**) Percentage of lymphocytes in fish exposed to oscillating (oscill.) or sustained (sust.) concentrations of BDP for 21 days (n = 30–36). (**b**) Percentage of precursor cells and (**c**) granulocytes in fish exposed to sustained concentrations of BDP (1 and 60 ng·h/mL) for 21 days (n = 30–36). (**d–f**) Representative forward scatter (FSC) versus side scatter (SSC) profile of white blood cells in control fish and in fish exposed to sustained concentrations (1 and 60 ng·h/mL) of BDP for 21 days. Note the concentration-dependent changes in the components of white blood cells populations. (**g,h**). Best-fit regression curves for GR gene expression (solid black curve) and decrease of lymphocyte (lymphocytopenia, solid red curve) expressed as effect magnitude at increasing plasma concentrations of BDP (expressed as AUC). Note the changes in the two curves in conditions of oscillating versus sustained exposure dynamics.

Long-term exposure to synthetic glucocorticoids in humans can lead to immunodepression. To investigate this AOP, we analysed white blood cells populations by flow-cytometry. In Experiment 1, the percentage of lymphocytes was significantly reduced after oscillating exposure to 1000 ng BDP/L for 21-days (*p* < 0.001) (Fig. 3a). The average reduction compared to control fish was 12 ± 16%. The severity of this effect was dramatically enhanced when exposure to BDP was sustained. In Experiment 2, effect concentration was significantly reduced compared to Experiment 1, with lymphocyte count decreased by 64 ± 13% after exposure to 10 ng BDP/L (*p* < 0.001) and by 73 ± 12% after exposure to 1000 ng BDP/L. Additionally, a significant increase of precursor cells was observed in both treatment groups (*p* < 0.001) (Fig. 3b), whereas only fish exposed to 1000 ng BDP/L showed hyper-granulocytosis (*p* < 0.001) (Fig. 3c). The latter effect was observed only after sustained exposure to the highest concentration of BDP and was never observed in any of the fish exposed to the oscillating concentrations of the drug. The typical response of the white blood cells population in the different treatments is represented in Fig. 3d–f. The magnitude of the phenotypic effect was not predicted by the magnitude of the transcriptional response of GR (Fig. 3g,h). No significant changes were observed in the gene expression of IL6 and TNFα in the liver (Supplementary Fig. S6).

### AOP: androgenic effects

**Figure 4:**
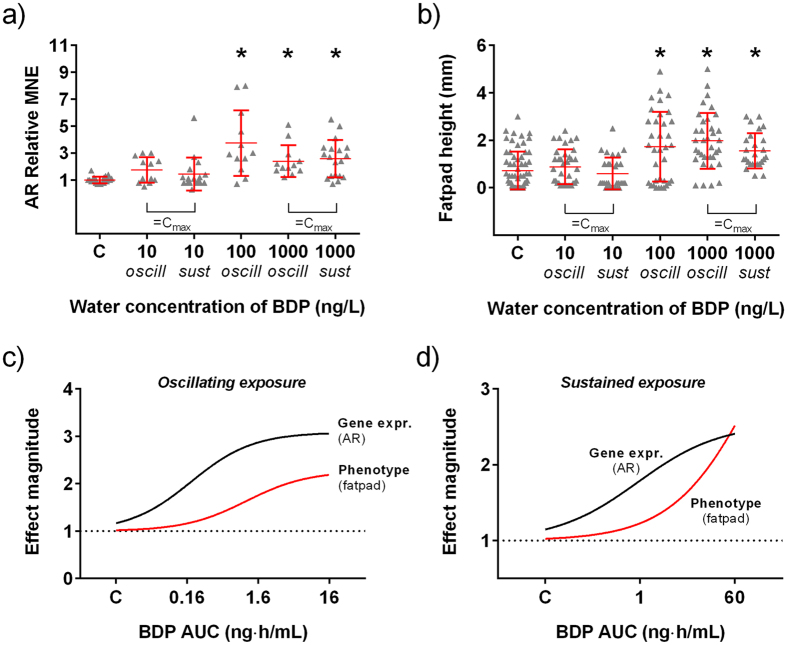
Androgenic effect of BDP on fathead minnows. (**a**) Relative Mean Normalized Expression (MNE) levels of AR measured in the liver of fish exposed to increasing plasma concentrations of BDP over 21 days (n = 12–20) and (**b**) fatpad height (n = 36) in the same fish. Fatpad is an androgen-dependent secondary sexual characteristic typical of fathead minnow males. Triangles indicate individual fish, red lines mean ± SD, and asterisks a significant difference versus the control group (p < 0.05). (**c,d**) These data were used to generate the best-fit regression curves for AR gene expression (solid black curve) and the fatpad height (solid red curve) expressed as effect magnitude at increasing plasma concentrations of BDP (expressed as AUC) in conditions of oscillating and sustained exposure to BDP.

The androgen receptor (AR) is a secondary-target of 17-BMP and its modulation can lead to skin androgenisation in humans. To investigate the androgenic effects of the drug, we quantified AR gene expression and a set of AR-mediated phenotypic responses (i.e. expression of secondary sexual characteristics, SSCs). Fathead minnow is a gonochoristic and sexually dimorphic species in which sexually mature males display specific SSCs, including nuptial tubercles on the snout and prominent fatpad on the dorsal area of the body, between the head and the dorsal fin. The degree of expression of these SSCs is modulated by androgens via the AR^13^, providing therefore an ideal endpoint to detect androgenic effects of chemicals. AR expression in the liver was significantly induced (*p* = 0.001) after oscillating exposure to 100 and 1000 ng BDP/L in Experiment 1 (Fig. 4a). The maximum observed expression induction was 8-fold. No significant effects were observed in fish exposed at 10 ng BDP/L. These results were also confirmed in Experiment 2, in which sustained exposure to 1000, but not to 10, ng BDP/L significantly induced AR (*p* = 0.002). Exposure to BDP caused the induction of male SSCs in both experiments, confirming the androgenic effects of BDP. Among SSCs, the number of nuptial tubercles was the most sensitive endpoint, which was significantly increased in fish exposed to both oscillating (*p* = 0.022) and sustained (*p* < 0.001) exposure to 10 ng BDP/L (Supplementary Fig. S7). Tubercle grade was increased after sustained exposure to 1000 ng BDP/L (*p* = 0.02), but not after oscillating exposure to the same nominal concentration (Supplementary Fig. S7). Fatpad height was increased by exposure to both oscillating (*p* < 0.001) and sustained (*p* < 0.001) exposure to 1000 ng BDP/L; additionally, in Experiment 1, fatpad height was increased after oscillating exposure to 100 ng BDP/L (Fig. 4).

### qAOP development

**Figure 5:**
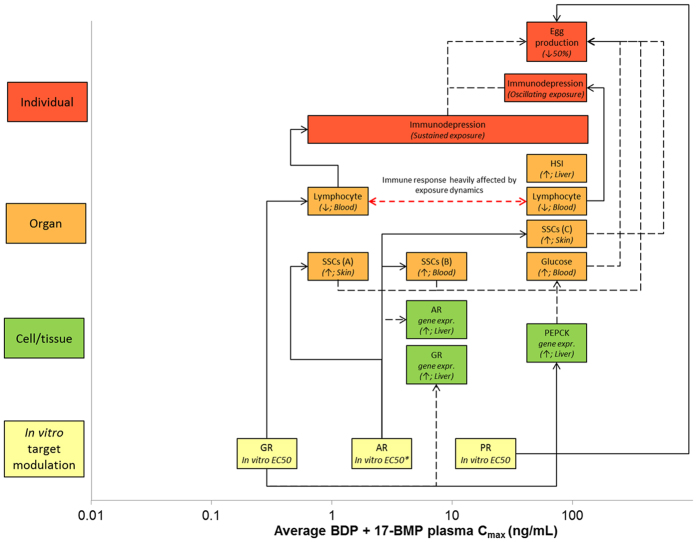
BDP-specific qAOP developed for adult fathead minnows exposed for 21 days and based on plasma drug concentrations. This qAOP portraits the link between the potency of 17-BMP (BDP active metabolite) to modulate GR (primary target), AR and PR (secondary targets) (expressed as *in vitro* EC50) and the *in vivo* effects observed on Mode-of-Action related endpoints at different levels of biological organisation. Each box is plotted against the lowest average drug plasma concentration at which the effect was observed. For each box at Cell/Tissue and Organ level, the identity of the tissue and organ in which the effect was quantified is provided. Arrows indicate the direction of the effect (increase or decrease). Only for the apical effect “Egg production” the magnitude of the effect is provided for quantitative purposes. For each box a dose response curve has been generated and is available in Supplementary Fig. S8. Full lines indicate known cause-effect linkages, whereas dashed lines indicate hypothesised linkages or linkages for which the direction of the response is difficult to predict or generalise (i.e. GR and AR gene expression). The asterisk indicates that the EC50 value for the modulation of AR refers to BOH, as no data are available for 17-BMP. Note that the immune response is highly dynamic and dependent on the dynamic of exposure.

The results of the present studies were integrated with the data produced in previous studies^12^,^14^. A BDP-specific qAOP network was generated to portray the quantitative link between the potency of 17-BMP (BDP active metabolite) to modulate GR (primary target), AR and PR (secondary targets) (expressed as *in vitro* EC50) and the *in vivo* effects observed on Mode-of-Action related endpoints at different levels of biological organisation (Fig. 5). These qAOPs explicitly refer to adult male fathead minnows exposed to BDP for 21 days. While some of the key events of the AOPs were relatively independent of the exposure dynamic (oscillating *vs* sustained), others (i.e. immunodepression) were profoundly affected. Considering the ability of BDP to modulate multiple molecular targets, the qAOP network developed here highlighted that multiple pathways likely converged to determine the apical effect (i.e. reproductive performance).

### BDP *versus* dexamethasone

To highlight the role of physico-chemical properties and PK/PD profile in driving the development of qAOP, we compared the effects induced by BDP with those induced by dexamethasone (DEX)^15^. Both glucocorticoids are considered to be very potent GR modulators. DEX was shown to have approximately 2-fold higher GR binding affinity than BDP and 13-fold lower than 17-BMP (active metabolite) *in vitro*^11^. However, after waterborne exposure, very high concentrations of DEX (50–500 μg/L) were required to elicit observable effects in fish (Table 1)^15^. This is a striking difference when compared with the 10 ng/L required for BDP. We hypothesised that the wide gap in the *in vivo* risk (500 to 50,000-fold) can be explained by the different physicochemical properties of the two compounds, which in turn translate into different uptake (from water to blood) and PK profiles (Table 1). For example, the Log D_7.4_ of BDP is ~100-fold higher than the one of DEX (4.07 *vs* 1.92, ACD prediction). This difference is 4- to 7-fold when volume of distribution (V_d_) is considered (17-BMP: 424 L *vs* DEX: 61–112 L)^11^,^16^,^17^.

## Discussion

Predictive toxicology models, including AOPs, are not intended to portray biological processes in all their complexity, but instead should be fit-for-purpose. The main challenge is to identify the minimum degree of complexity required to achieve the desired predictive power in a given decision-making context. Several approaches have been proposed to incorporate complexity into AOPs, from the application of systems biology modelling to the creation of AOP networks^18^,^19^,^20^. Here, using synthetic glucocorticoids as model chemicals, we showed that exposure dynamics, internal exposure and chemical-specific PK/PD properties have profound influences not only on the application of a given AOP, but also on AOP development and its potential transferability across chemicals in a decision-making context.

Responses of biological endpoints to chemicals are highly dynamic and dependent on dose and time. In the present study we showed that different pathways can have different sensitivities to exposure dynamics. The knowledge of pathway-specific response dynamics is therefore important to enhance the power of AOPs to predict effect magnitude and degree of adversity. The comparison of the same set of MoA-related endpoints in conditions of oscillating or sustained chronic exposure indicated that, with the exception of immune response, the response of the selected endpoints (GR, AR, PEPCK gene expression; SSCs) was mainly driven by the C_max_. Once the responses were induced, their magnitude was not affected by increasing AUCs. It is important to highlight that this observation refers to adult male fish, and its validity for adult females or other life stages is unknown. It is possible that, in our study, inter-individual variability, sex- and life-stage specific margins of response at both molecular and physical level acted in combination to hide the effect of different AUCs on the response magnitude. For example, nuptial tubercles are normally expressed in adult male fathead minnows and the magnitude of their induction will be limited by the skin surface available for their development and by the physical limitations to their growth. The use of juveniles or females, that do not express nuptial tubercles, would likely offer a wider margin of response and increase the sensitivity of the observation. Notably, in the case of the immune response, the LOEC (expressed as AUC) in conditions of sustained exposure was approximately 37-fold lower than the one observed after oscillating exposure. This concentration gap is likely to be even higher as no NOEC was observed. The difference in the response of WBCs was not only quantitative, but also qualitative. Sustained exposure to 1000 ng BDP/L led to the decrease of circulating lymphocytes as well as to a significant increase of granulocytes. The latter effect was never observed in any fish exposed to oscillating concentrations of BDP. This suggests that the specific temporal dynamic modulation (i.e. continuous over 21 days) of the GR was the major driver of the magnitude of the response rather than the drug concentration alone. Overall, these results indicate that in some cases, effect concentration values (e.g. LOECs) are not static and can vary greatly in different exposure scenarios. Considering the importance of exposure dynamics to determine magnitude and adversity of effects^21^,^22^,^23^,^24^, determining the pathway-specific sensitivity to this factor can increase the predictive power of the qAOP.

Establishing quantitative relationships between chemical-induced perturbations at molecular level and their manifestation at higher level of biological organization (tissue/organ/organism) is one of the main challenges for safety pharmacology and toxicology^25^,^26^. The endpoints quantified in the present studies were selected on the basis of the known MoA of BDP in humans and expression of specific genes was anchored to related phenotypic endpoints. Each endpoint could therefore be considered a Key Event of the AOP. Considering the importance of characterizing the predictive value of responses at low level of organization, we compared dose-response curves of inter-dependent endpoints (gene expression/phenotype) in order to investigate whether, after chronic exposure, MoA-specific molecular responses occur also at drug concentrations that do not cause observable phenotypic responses. In some cases changes of gene expression were shown to be more sensitive than phenotypic responses (i.e. responding at lower concentrations; PEPCK, GR), but in other cases they were not (AR, IL6, TNFa). Additionally, except for PEPCK, the magnitude of gene expression responses was not predictive of the magnitude of the associated phenotypic effect. Some caution is required for the interpretation of these results as gene expression was quantified only in the liver whereas the phenotypic responses were measured elsewhere. The analysis of the pair PEPCK/blood glucose carries the highest degree of confidence as the two endpoints were analysed in the most relevant tissues (liver, the main site of gluconeogenesis, and blood). However, facial derma and hematopoietic tissues would have been the most relevant tissues for, respectively, AR and IL6/TNFa gene expression. In the context of qAOP development and predictive toxicology, these considerations highlight the importance of tissue relevance and imply that dedicated study-designs are necessary to unravel the complex link between molecular responses and effect at higher phenotypic level^27^. For example, Andersen *et al.* (2008)^28^ investigated the correlation of histological and gene expression changes in the nose of rats exposed to formaldehyde. In that case genomic changes did not prove to be more sensitive than tissue responses. In contrast, after exposure of zebrafish to progesterone^29^ significant changes in the expression of genes involved in ovarian physiology were observed at concentrations approximately 90-fold lower than the ones required to induce observable histopathological effects. Despite the utility of transcriptomic approaches in investigative toxicology, the informative power of gene expression profiling as a stand-alone approach in decision-making remains to be clarified^27^,^30^. Anchoring these responses to adverse phenotypes arising after chronic exposure is still a critical factor for data interpretation.

Transferability of AOP across chemicals is one of the main theoretical advantages of the AOP concept, as it could allow a cost-effective, reliable and faster risk assessment. Nevertheless, a theoretical challenge arises when chemicals with a significant polypharmacology profile (e.g. some pharmaceuticals) are used as a reference compounds in the AOP development. For example, BDP is known to modulate GR, AR, and PR. The perturbation of these three different pathways can converge toward apical endpoints, such as reproductive activity. Specifically, in the present studies, it can be hypothesised that increased levels of plasma glucose, immunodepression, androgenic effects and hormonal feedbacks all contributed to reduce reproductive performance. To what extent each individual pathway contributed to the final apical effect is unknown. If the qAOP generated for BDP is used to predict the risk for a different GR agonist with a different polypharmacology profile (e.g. with no AR and PR agonistic activity), the quantitative prediction of reproductive effects may not be accurate. This indicates that chemical-specific polypharmacology profiles need to be explicitly taken into account during qAOP development in order to enable an appropriate application of qAOP to different chemicals. In this context, the increasing availability of *in vitro* activity data generated by the ongoing chemical toxicity screening programs (e.g. US EPA’s Toxcast) together with the concept of AOP network proposed by Knapen *et al.* (2015)^31^ would provide valuable tools to address the challenge of polypharmacology and increase the predictive power of qAOPs.

The AOP concept embraces the vision that mechanistic information (e.g. bioactivity profiles) acquired through *in vitro* assays for reference chemicals can be used to predict the risk of similarly acting chemicals. The potency of a given chemical to modulate one or more targets *in vitro* could therefore drive risk assessment. For example, once the qAOP for GR agonists is developed using a model GR agonist, the risk of other GR agonists could be predicted on the basis of their *in vitro* potency on the GR receptor. To test this assumption we compared the results obtained in our studies with the effects induced by another model glucocorticoid, dexamethasone, in fathead minnow^15^. *In vitro*, 17-BMP (the active metabolite of BDP) is 13-fold more potent than DEX to modulate the GR; however, once tested *in vivo*, BDP was 500 to 50,000-fold more potent to induce the same endpoints as DEX in a very similar exposure set-up. This discrepancy may well be explained by the chemical-specific properties and by the chemical-specific PK profile of the two compounds. For example, BDP has a higher Log D_7.4_ and higher V_D_ than DEX. The higher Log D_7.4_ suggests a higher uptake from water into blood, whereas the high V_D_ of BDP suggests that after administration the drug readily diffuses in the organism and efficiently enters the organs, whereas the lower V_D_ of DEX suggests that the drug tends to remain more in the blood compartment. During their intended use in humans, these differences are accounted for in the administration route (e.g. intravenous injection *vs* inhalation) and therapeutic use (e.g. different dosing regimen). However, in our case, given the same exposure route in all the experiments (waterborne), the differences in uptake and PK profile were likely to be the major factors to explain the different *in vivo* potencies and toxicological profiles of the two compounds. In our studies, we demonstrated that BDP is readily taken up by fish and converted into 17-BMP and BOH, as predicted by the knowledge of the drug metabolism in humans. Plasma concentrations of DEX were not quantified in the study performed by LaLone *et al.* (2012)^15^. Using the FPM, the water concentration of DEX predicted to elicit therapeutic effects in fish is in the range 2,000–12,000 ng/L; whereas, for BDP the same values is between 6 and 30 ng/L. These basic predictions, solely driven by the partitioning factor of the chemicals (i.e. LogK_OW_ or Log D_7.4_), can already explain to a large extent the observed differences in the LOECs of the two compounds. It is important to note that the predictions are referred to therapeutic effects extrapolated from human data and that adverse effects are predicted to occur at higher drug plasma concentrations or with the increase of treatment duration. This point is particularly important as the phenotypic endpoints measured in our studies and in the studies conducted by LaLone *et al.* (2012)^15^ can be considered as adverse responses. Using the FPM, the predicted DEX plasma concentration in fish that exhibited adverse effects was approximately 1400 ng/mL (based on mean measured water concentrations). These values would be 40- to 175-higher than the human therapeutic concentration range (8–35 ng/mL)^32^,^33^, whereas plasma concentrations of BDP in fish that exhibited adverse effects were 1 to 50-fold higher than the blood concentrations measured in humans after BDP administration via inhalation.The example of BDP and DEX clearly indicate that chemical-specific properties should be carefully considered in the studies performed with the aim to develop qAOPs and in the selection of reference chemicals. In fact, similarly potent compounds with different uptake and PK profiles may exhibit different *in vivo* risks. This discrepancy can be predicted by uptake and PK/PD considerations.

Overall the theoretical observations and the experimental evidence provided in this study suggest that the consideration of species-species uptake (for wildlife) and physiologically-based PK/PD models during the development of qAOPs can significantly enhance their predictive power, enabling a more accurate assessment of the risk and the reliable transferability of qAOPs across chemicals. In this study we used human data to predict and interpret effects in fish (Read-Across approach)^34^. This extrapolation exercise can be highly predictive^35^, but its success is critically dependent on the accurate extrapolation of dosimetry. The development of more accurate physiologically-based PK/PD models able to tackle chronic exposure scenarios for different model species will empower cross-species extrapolation and allow us to maximise the informative and predictive power of data generated using different models.

## Materials and Methods

### Ethics statement

All the experimental *in vivo* protocols and procedures involving fish were performed in accordance with the United Kingdom Animals (Scientific Procedures) Act. The *in vivo* studies were carried out at Brunel University London (United Kingdom) under Project License and Personal Licences granted and approved by the United Kingdom Home Office. All *in vivo* experimental protocols were also approved by the Research Ethics Committee of Brunel University London.

### Characterization of chemical exposure

Several studies were conducted to elucidate the chemical behaviour of the test chemical BDP in a laboratory-based flow-through exposure system used to expose fish to several concentrations of BDP for 21 days (Supplementary Fig. S2). LC-MS/MS was used to assess the stability of BDP, 17-BMP and BOH in both water and solvent. The methodological details of the studies and the results are provided in the Supplementary Information and in Supplementary Table S1, Supplementary Table S2, and Supplementary Fig. S3. The concentrations of the three chemicals were monitored at several time points during the *in vivo* studies (Supplementary Fig. S4, Supplementary Table S3). Additionally, an in *in vitro* GR assay was used to evaluate if degradation products of BDP, 17-BMP and BOH retained or not pharmacological activity (Supplementary Fig. S3).

### *In vivo* exposures

Two independent 21-day *in vivo* experiments were conducted using a continuous flow-through system as described in the SI. Experiment 1 was characterized by oscillatory concentrations of BDP reaching their peak (equal to the nominal concentration) every 4 days, whereas fish in Experiment 2 were exposed to sustained concentrations of the drug over the entire duration of the study. The two studies had the same peak concentrations (C_max_) of BDP but different time-integrated concentrations (AUCs). The details of the study design and of the chemicals used are provided in the Supplementary Information. Experiment 1 included three treatment groups exposed to nominal concentrations of 10, 100 and 1000 ng BDP/L and one control group receiving only clean water. Each treatment group included three replicate tanks, each hosting 12 adult male fathead minnows (36 fish per treatment). Experiment 2 included two treatment groups exposed to 10 and 1000 ng BDP/L and one solvent control group. Water in all fish tanks contained DMF at 0.0095%, which is within the limit recommended by OECD guidelines. Each treatment group included three replicate tanks, each hosting 12 adult male fathead minnows (36 fish per treatment).

In Experiment 1, the selection of test water concentrations was driven by the application of the Fish Plasma Model^10^,^35^, which predicted resulting fish plasma concentrations respectively equal (group: 10 ng/L) and above (groups: 100, 1000 ng/L) the Human Therapeutic Concentrations (H_T_PCs) range of the active metabolite 17-BMP. This therapeutic range was retrieved from a clinical trial in which patients were treated with repeated administrations of different doses of BDP (80, 160, 320 μg/day) via inhalation^36^. The observed range of therapeutic C_max_ of 17-BMP was between 0.8 and 1.4 ng/mL. It is important to note that these concentrations reflect the systemic level of 17-BMP after inhalation. It is likely that concentrations of 17-BMP in the upper respiratory tract are higher than the systemic concentrations in the blood. Nevertheless, blood concentrations are highly relevant for the prediction of adverse effects at systemic level. In Experiment 2, the first exposure concentration (10 ng/L) was selected as it was the NOEC of Experiment 1. The aim was to evaluate if the NOEC was constant under different exposure dynamics. The second concentration (1000 ng/L) induced statistically observable effects in the majority of measured endpoints. The aim was to evaluate if different exposure dynamics are able to affect the magnitude of the effects, and if yes, to what extent. From a PKPD standpoint, the question was whether the response of each endpoint was driven by the C_max_ or by the AUC.

### Sampling and analysis of endpoints

After 21 days of exposure to BDP, fish were terminally anaesthetised using ethyl 3-aminobenzoate methanesulfonate salt (MS-222, 0.5 g/L; adjusted to pH 7.5 with 1 M NaOH) (Sigma, Poole, UK; CAS No: 144-55-8). Weight and fork length were measured, and these parameters were used to calculate the condition index (CI). Blood was collected from each fish via the caudal peduncle using 75 μL heparinised capillary tubes, decanted into eppendorfs and immediately centrifuged at 7000 × g for 5 min at 4 °C. Plasma was collected and stored at −80 °C until analytical quantification of BDP, 17-BMP and BOH by LC-MS/MS following the method detailed in the SI. After plasma separation, blood cells were reconstituted with 200 μL of Hank’s balanced salt solution (HBSS, Sigma), and the white blood cells (WBC) were isolated, stained with DiOC6 (3,3′-dihexyloxacarbocyanine iodide) and analysed using flow cytometry (BD FACS Canto II) as described by Kugathas *et al.* (2013)^12^. Three main groups of cells were identified and quantified using the FlowJo software package (Treestar, Ashland, OR): lymphocytes, white blood cells precursors, and granulocytes, as described by Page *et al.* (2013)^37^ (*n* = 50,000) (Supplementary Fig. S5).

After blood collection, liver was immediately removed for molecular analyses and stored at −80 °C until RNA extraction. Its weight was used to calculate the hepatosomatic index (HSI = liver weight (g)/body weight (g) × 100). Finally, the expression of secondary sexual characteristics (SSCs) was recorded for every fish as described by Margiotta-Casaluci *et al.* (2013)^13^.

Total RNA was isolated from individual liver samples using the RNeasy Midi Kit (Qiagen), according to the manufacturer’s protocol. Quantity and purity of each RNA sample were determined by spectrophotometry (Nanodrop, Fisher Scientific), and RNA integrity was visually checked by agarose gel electrophoresis. Complementary DNA (cDNA) was synthesised from 1 μg total RNA using Invitrogen SuperScript III First-Strand Synthesis System for reverse transcription-PCR kit according to the manufacturer’s protocol. Gene expression of ribosomal protein L8 (rpl8, house-keeping gene), GR, androgen receptor (AR), phosphoenolpyruvate carboxykinase (PEPCK), interleukin-6 (IL6) and tumor necrosis factor alpha (TNFα) was quantified by qPCR. Primers for the listed target genes are provided in Supplementary Table S4. All primers were validated and optimised for annealing temperature before sample analysis. qPCRs were performed in triplicate on cDNA from individual fish using a CFX96 Real-Time PCR detection system (Bio-Rad) and Fast SYBR Green Master Mix (Invitrogen) according to the manufacturer’s protocol. The relative expression of the target genes were normalized to the expression of the housekeeping gene using the Excel-based software qGENE^38^, which takes into account the amplification efficiency of both the target genes and the reference gene to calculate the mean normalized expression (MNE) of each target gene.

### Prediction of drug plasma concentrations

Measured plasma concentrations were compared to the concentrations predicted by the Fish Plasma Model (FPM) as described by Hugget *et al.* (2003)^10^ and Margiotta-Casaluci *et al.* (2014)^35^. The Log K_OW_ and the Log D_7.4_ used in the model were predicted by ALOGPS and were, respectively, 3.69 and 4.07. The predictions of the FPM were based on water concentrations measured on Day 21. Water concentrations measured at different time points during the studies were used to predict drug plasma concentrations over 21 days, expressed as C_max_ and AUC values. The latter was calculated using the GraphPad Prism Software (GraphPad Software, Inc., US).

### Statistical analysis

Statistical analyses were conducted using SigmaStat software (version 3.5, Systat Software Inc., Germany). Data were analysed for normality (Kolmogorov–Smirnov test) and variance homogeneity (Levene’s test). Where assumptions of normality and homogeneity were met, one-way analysis of variance (ANOVA) was followed by the Dunnett’s test to compare the treatment means with respective controls. Where the assumptions were not met, data were analysed using a non-parametric test, Kruskal–Wallis ANOVA on Ranks, followed by Dunn’s post hoc test^39^. Statistical significance was set at a level of *p* < 0.05, unless otherwise indicated.

### qAOP development

The results obtained in the present studies were integrated with those obtained by Kugathas *et al.* (2013)^15^, who performed a 21-day *in vivo* exposure to BDP using fathead minnow and the same flow-through exposure set-up described for Experiment 1. Specifically, the data used from that study concerned GR and PEPCK gene expression in liver samples and blood glucose concentrations. Effect water concentration for egg production was provided by Thrupp *et al.* (2015)^14^, who performed a 21-day fathead minnow pair breeding assay in flow-through conditions similar to the ones described for Experiment 1. All the measured endpoints were used to develop a BDP-specific qAOP based on drug plasma concentrations. Additional data on the effects of BDP in fish were retrieved from Carney Almroth *et al.* (2015)^40^, who exposed rainbow trout for 14-day in flow-through conditions. The latter data were not used to generate the graphic AOP as they are based on rainbow trout rather than fathead minnow, but are provided in Supplementary Table S5. *In vitro* EC50 data for the modulation of GR (primary target), AR and PR (secondary targets) were retrieved from the literature. However, the vast majority of available data referred to either BDP or BOH, but not to 17-BMP, which is the active metabolite. The used EC50 s for 17-BMP referred to 1) GR transactivation assay and coactivator recruitment assay^41^ (median EC50 value = 0.9 nM = 0.4 ng/mL) 2) PR binding assay (EC50 = 50 nM = 23 ng/mL)^42^. No data were available for the *in vitro* modulation of AR by 17-BMP, thus the EC50 for BOH was used (EC50 = 12 nM = 5 ng/mL; AR beta-lactamase assay in agonist mode; Pubchem ID 20469).

## Additional Information

**How to cite this article**: Margiotta-Casaluci, L. *et al.* Internal exposure dynamics drive the Adverse Outcome Pathways of synthetic glucocorticoids in fish. *Sci. Rep.* **6**, 21978; doi: 10.1038/srep21978 (2016).

## Supplementary Material

Supplementary Information

## Figures and Tables

**Table 1:** Comparison of relevant PK properties, *in vitro* and *in vivo* potencies of beclomethasone dipropionate and dexamethasone.

	BECLOMETHASONE DIPROPIONATE	DEXAMETHASONE	References
	*PHYSICOCHEMICAL & PHARMACOKINETIC PROPERTIES*		
*CAS No.*	5534-09-8	50-02-2	
*Log K*_ow_*	3.69	1.87	
*Log D*_7.4_*	4.07	1.92	
*V*_*D*_*(L) (i.v. administration)*	20 (BDP)	61–112	11, 16, 17
	424 (17-BMP)		
*Plasma t*_*1/2*_ *(h)*	0.5 (BDP)	3.35	16
	2.5 (17-BMP)		
*Predicted BCF*_*water:blood*_ *(fish)*	65–124	3	
	*IN VITRO POTENCY*		
*In vitro relative affinity to GR (relative to DEX)*	53 (BDP)	100	11
	1345 (17-BMP)		
	*EFFECTS IN FISH (LOEC, ng/L)*		
*GR gene expression*	100	50,000	Present study; 15
*In vivo SSCs*	10	>500,000	
*In vivo reproduction*	1000	500,000	
