# Corrigendum: Internal exposure dynamics drive the Adverse Outcome Pathways of synthetic glucocorticoids in fish

**DOI:** 10.1038/srep28122

**Published:** 2016-06-20

**Authors:** Luigi Margiotta-Casaluci, Stewart F. Owen, Belinda Huerta, Sara Rodríguez-Mozaz, Subramanian Kugathas, Damià Barceló, Mariann Rand-Weaver, John P. Sumpter

Scientific Reports 6: Article number: 2197810.1038/srep21978; published online: 02262016; updated: 06202016

The original version of this Article contained typographical errors.

In the Abstract,

“We recommend that the development phase of qAOPs should include the application of species-species uptake and physiologically-based PK/PD models.”

now reads:

“We recommend that the development phase of qAOPs should include the application of species-specific uptake and physiologically-based PK/PD models.”

In the Discussion section,

“Overall the theoretical observations and the experimental evidence provided in this study suggest that the consideration of species-species uptake (for wildlife) and physiologically-based PK/PD models during the development of qAOPs can significantly enhance their predictive power, enabling a more accurate assessment of the risk and the reliable transferability of qAOPs across chemicals.”

now reads:

“Overall the theoretical observations and the experimental evidence provided in this study suggest that the consideration of species-specific uptake (for wildlife) and physiologically-based PK/PD models during the development of qAOPs can significantly enhance their predictive power, enabling a more accurate assessment of the risk and the reliable transferability of qAOPs across chemicals.”

These errors have now been corrected in the PDF and HTML versions of the Article.

