# Supplementary material for: Internal exposure dynamics drive the Adverse Outcome Pathways of synthetic glucocorticoids in fish
Source: Sci Rep. 2016 Feb 26;6:21978. doi: 10.1038/srep21978 (PMC4768075; doi:10.1038/srep21978)
Supplement: Supplementary Information [file srep21978-s1.pdf]

Supplementary Information

Internal exposure dynamics drive the Adverse Outcome Pathways of synthetic glucocorticoids in fish

Luigi Margiotta-Casaluci[1,2,*], Stewart F. Owen[2], Belinda Huerta[3], Sara Rodríguez-Mozaz[3], Subramanian Kugathas[1], Damià Barceló[3,4], Mariann Rand-Weaver[5], John P. Sumpter[1]

[1] Brunel University London, Institute of Environment, Health and Societies, London, UB8 3PH, United Kingdom

[2] AstraZeneca, Global Environment, Alderley Park, Macclesfield, SK10 4TF, United Kingdom

[3] Catalan Institute for Water Research (ICRA), Scientific and Technological Park of the University of Girona, Girona, 17003, Spain

[4] Water and Soil Quality Research Group, Department of Environmental Chemistry, IDAEA-CSIC, Jordi Girona 18-26, 08034 Barcelona, Spain

[5] Brunel University London, College of Health and Life Sciences, London, UB8 3PH, United Kingdom

* **Corresponding author**: Email: Luigi.Margiotta-Casaluci@brunel.ac.uk; Phone: +44 (0)1895 268796; Address: Brunel University London, Institute of Environment, Health and Societies, Kingston Lane, UB8 3PH, London, UK.

CONTENT

Chemistry Section (p. 3-15)

Characterization of chemical exposure

Rationale

Materials and methods

Results

Figure S1. Chemical structures of BDP and three of its transformation products.

Figure S2. Scheme of flow-through exposure system used for ecotoxicological studies.

Figure S3. Stability of BDP, 17-BMP and BOP and pharmacological activity.

Figure S4. Water concentrations of BDP in Experiment 1 and Experiment 2.

Table S1. SRM transitions and compound dependant MS parameters for the target analytes.

Table S2. Stability of BDP, 17-BMP and BOH (1 µg/L) under different storage conditions.

Table S3. Concentrations of BDP in water samples collected during the in vivo exposure studies.

Biology Section (p. 16-22)

Figure S5. White blood cells scatter and gating.

Figure S6. Gene expression panels.

Figure S7. Effect of BDP on fish sexual secondary characteristics.

Figure S8. Concentration response-curve for the endpoints measured in Experiment 1 and 2.

Table S4. Primer sequences used for qPCR reactions.

Table S5. Detailed list of effects observed in fish after exposure to BDP and dexamethasone.

CHEMISTRY SECTION

CHARACTERIZATION OF CHEMICAL EXPOSURE

Study rationale

Exposure of fish to beclomethasone dipropionate (BDP) for 21 days in Experiment 1 caused mode-of-action driven effects at nominal concentrations ≥100 ng/L (i.e. hyperglycaemia, skin androgenisation, decreased lymphocyte population); however, unexpected time-dependent drug stability problems identified subsequently prevented an exact determination of NOEC and LOEC values. Due to the observed *in vivo* potency of BDP, we then performed a number of studies to characterize the chemical behaviour of BDP in the flow-through exposure system used in Experiment 1. The results of these studies showed an highly reproducible degradation dynamics of BDP in water that allowed a retrospective reliable determination of exposure dynamics in Experiment 1. Finally, the same results were used to drive the design of Experiment 2 and aid the interpretation of the results of our previous exposure experiments (12).

Materials and methods

Chemicals and dilution water

High purity grade (>99%) BDP (CAS n° 5534-09-8) and BOH (CAS n°: 4419-39-0) were purchased from Sigma-Aldrich. 17-BMP (CAS n°: 5534-18-9) was purchased from Steraloids, Inc. Isotopically labelled BDP-d10, used as internal standard (IS), was acquired from Toronto Research Chemicals. Dechlorinated tap water (5 and 10 μm carbon filtered) was used as dilution water, and general parameters (pH, temperature and dissolved oxygen) were monitored daily throughout the study. Water pH ranged from 7.3 to 7.7, temperature from 24.5 to 25.7 °C, and dissolved oxygen from 7.2 to 7.9 mg/L.

Flow-through exposure system

Two 21-day *in vivo* exposure studies were carried out at Brunel University London using a flow-through system. A generalised scheme of the system is represented in Figure S2. Thermostatically heated (25±1°C) dechlorinated tap water flowed into individual glass mixing chambers (12 in Experiment 1; 9 in Experiment 2) at a rate of 200 mL/min. The same chambers also received the stock solution of the test chemical via peristaltic pump at a rate of 0.2 mL/min (Experiment 1) and 0.02 mL/min (Experiment 2), in order to achieve the desired nominal concentrations. From each mixing chamber water flowed into one glass tank (20 L) hosting 12 fish. Each fish tank underwent approximately volume changes per day. In Experiment 1, BDP concentrated stock solutions were prepared in double-distilled water every four days. In Experiment 2, BDP concentrated stock solutions were prepared in N,N-dimethylformamide (DMF; CAS number 68-12-2; ≥99%) (Sigma, Poole, UK) and replaced every seven days. Experiment 1 included three treatment groups exposed to nominal concentrations of 10, 100 and 1000 ng BDP/L and one control group receiving only clean water. Experiment 2 included two treatment groups exposed to 10 and 1000 ng BDP/L and one solvent control group. Water in all test vessels contained DMF at 0.0095%, within the limit recommended by OECD guidelines. Each treatment group included three replicate tanks. The stability of BDP and its two major metabolites, 17-BMP and BOH (Figure S1), was investigated both in concentrated solutions and in fish tanks, two critical sites of chemical transformation. To ensure that the observed chemical behaviour of BDP was not due to laboratory-specific conditions, the studies on BDP stability were replicated independently at Brunel University London (London, UK) and Catalan Institute of Water Research (Girona, Spain). Water samples from fish tanks were collected on Day 0, 4, 7, 14, and 21 and analysed by LC-MS/MS.

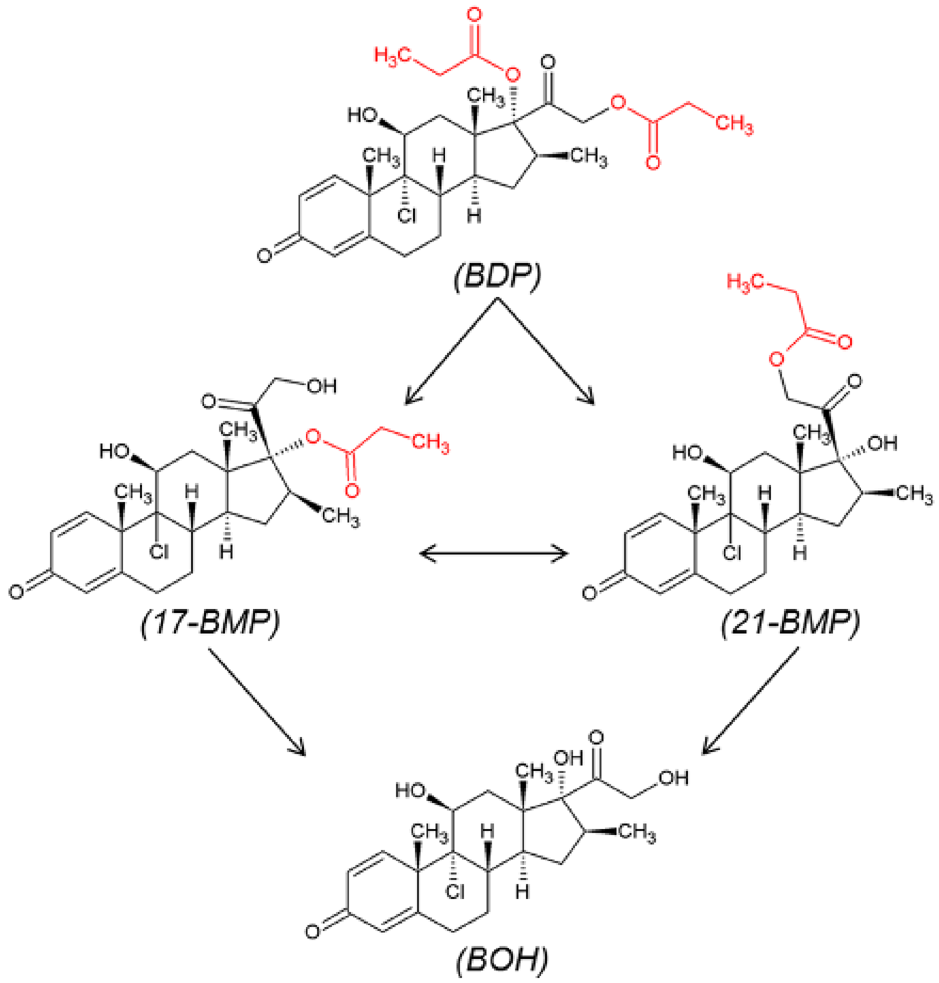

Figure S1. *Chemical structures of BDP and three of its transformation products. 17-BMP is the active metabolite. Propionate groups are indicated in red.*

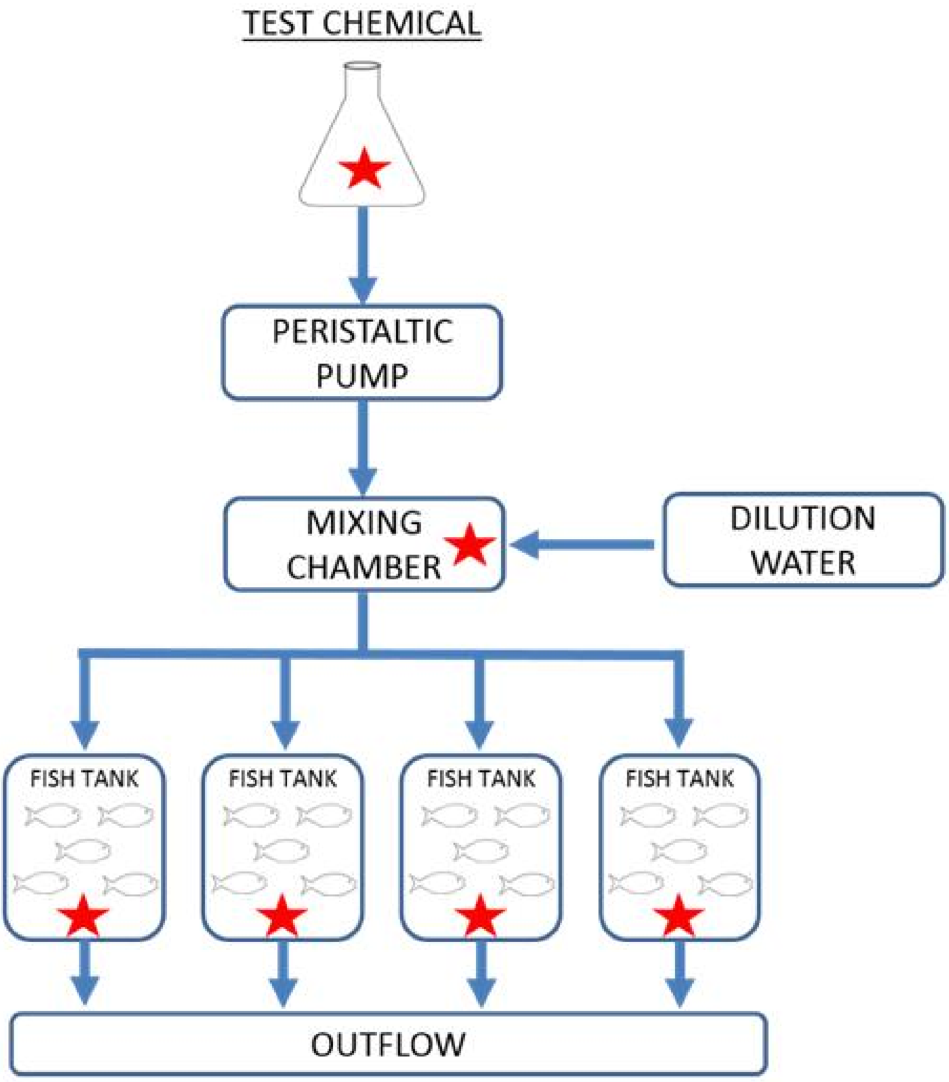

Figure S2. *Example of flow-through exposure system used for ecotoxicological studies. Typically the parent compound is used to make up concentrated stock solutions that are then delivered by high-precision peristaltic pumps or syringes to mixing chambers that also receive clean water. In those chambers the test compound is diluted to the desired concentration and reaches the final test vessels at a given flow rate with the aim to achieve the desired number of volume changes per day. In this type of set-up, unpredicted and/or unwanted chemical transformations may occur at different points along the system (red stars), such as in the stock solution container, in the mixing chamber or in the final exposure vessel.*

Stability studies.

The possible transformation of BDP in concentrated stock solutions was investigated by incubating concentrated BDP solutions (10,000 µg/L) prepared in different solvents (water and DMF) and at different pH (6.0, 7.4, 8.4) at 25°C for 96h in amber glass bottles. The concentrations of BDP and its potential transformation products (17-BMP, BOH) were quantified every 24h. Additional experiments were conducted to confirm the initial results and to investigate not only the stability of BDP, but also of 17-BMP and BOH. Concentrated stock solutions were prepared in different solvents (water, methanol, water/methanol (90:10, *v/v*) and incubated for 72h at 25°C. Samples were collected every 8 h and analysed immediately by LC-MS/MS. Additionally, the stability of BDP, 17-BMP and BOH was assessed in samples preserved under different conditions and analysed at different time points after collection, this being the most realistic scenario in ecotoxicology and environmental chemistry studies (43). Two sample storage strategies were employed during a period of 21 days using water samples collected from control fish tanks during an *in vivo* study. This water likely contained the typical load of biological material, such as bacteria and microalgae, associated with fish tanks, but did not contain any xenobiotic chemical other than the one spiked during the procedure. In the first strategy, water samples were spiked, separately, with 1 µg/L of BDP, 17-BMP and BOH and stored at −20°C in PET containers for up to 21 days. In the second preservation strategy, water samples were spiked with 1 µg/L of BDP, 17-BMP and BOH, loaded immediately onto solid phase extraction (SPE) cartridges and stored at −20°C. All samples were analysed on Day 0, 4, 14, and 21.

Pharmacological activity of transformation products.

To investigate if the transformation products generated in water retained pharmacological activity, the GR activity of water solutions of BDP, 17-BMP and BOH was quantified using a Human GR Reporter Assay System (Indigo Biosciences) following the manufacturer's instructions. Equipotent solutions of dexamethasone (DEX, used as reference compound), BDP, 17-BMP and BOH were prepared on the basis of their relative potencies (44, 45). The relative potency factors used were DEX

: BDP : 17-BMP : BOH = 1 : 0.4 : 13.5 : 0.8. BDP, 17-BMP, and BOH water solutions were assayed immediately after preparation (Time 0) and after 4 days of incubation at 25°C. At each time point, the concentrations of the test compounds were quantified by LC-MS/MS.

Preparation of water and plasma samples.

Samples collected from concentrated stock solutions and fish tanks were diluted with the appropriate volume of methanol (MeOH) or water to reach a MeOH:water ratio of 50:50 (*v/v*) and spiked with internal standard (BDP-d10, 10 µg/mL) before analysis by LC-MS/MS. SPE was applied to all the other samples (46). Extracts were reconstituted in 1 mL of methanol/water (50:50, *v/v*) and 10 µL of BDP-d10 solution (1 mg/mL). Concentrations of BDP, 17-BMP and BOH were quantified by LC-MS/MS.

Plasma samples (10-50 µl) were added to 400 µl of acetonitrile to achieve protein precipitation and vortexed for 15 s. Samples were transferred to an Ostro 96-well plate (Waters, USA) connected to a vacuum system for the removal of phospholipids. An aliquot of 300 µl was collected from each extract and was placed under a N2 current to dry completely. Finally, extracts were dissolved in 100 µl of methanol/water (1:1). Concentrations of BDP, 17-BMP and BOH were quantified by LC-MS/MS

Extractions efficiency for BDP, 17-BMP and BOH were, respectively, 82%, 70% and 100%. The method detection limits (MDL) and method quantification limits (MQL) were 0.003 and 0.009 ng/L for BDP, 0.007 and 0.020 ng/L for 17-BMP, and , 0.034 and 0.115 ng/L for BOH.

Chromatography and mass spectrometry analysis (LC-MS/MS)

Chromatographic separation was carried out with a Waters Acquity Ultra-PerformanceTM liquid chromatography system (UHPLC), using an Acquity HSS T3 colum (50 mm×2.1 mm i.d., 1.8 µm). Separation conditions were as follows: solvent (A) methanol, solvent (B) 10 mM formic acid/ammonium formate (pH 3.2) at a flow rate of 0.5 mL/min. The mobile phase gradient was programmed as follows: 0 min 40% A, 0.02–0.50 min 100% A, 0.5–3.5 min 100% A, 3.5–4.5 min

100–40% A, 4.5–5.5 min 40% A, a flow rate of 0.5 mL/min. The sample volume injected was 10 µL. The UHPLC instrument was coupled with a 5500 QTRAP hybrid triple quadrupole–linear ion trap mass spectrometer (Applied Biosystems) with an electrospray interface. Compound dependent MS parameters (declustering potential (DP), collision energy (CE) and collision cell exit potential (CXP)) as well as compound selected reaction monitoring (SRM) transitions were optimized by direct infusion of individual standard solution of each analyte at 10 µg/L (Table S1). All transitions were recorded in Scheduled MRM algorithm with 30 s detection window. Source dependent parameters were: curtain gas (CUR): 30 V; nitrogen collision gas (CAD): medium; source temperature: 300 °C; ion spray voltage: 5500 V; ion spray gases GS1: 60 V and GS2: 70 V. Instrument control data acquisition and data analysis were carried out using Analyst software (Applied Biosystem). Two SRM transitions between the precursor ion and the two most abundant fragment ions were monitored for each compound. The first transition was used for quantification purposes, whereas the second one was used to confirm the identity of the target compounds.

Table S1. *SRM transitions and compound dependant MS parameters for the target analytes.*

**Compound**	**Precursor ion (m/z)**	**MRM_1**	**MRM_2**	**RT (min)**	**DP**	**CE**	**CXP**
BOH	409 $[M+H]^+$	391	279	2.00	41	17	16
17-BMP	465 $[M+H]^+$	279	337	2.40	60	30	10
BDP	521 $[M+H]^+$	337	319	2.70	18	26	12
BDP-d_{10}	531 $[M+H]^+$	319	-	2.70	71	25	14

**RT: retention time; DP: declustering potential; CE: collision energy; CXP: collision cell exit potential.*

Results

Stability of concentrated solutions of BDP, 17-BMP and BOH in water and solvent.

The first set of experiments was conducted to assess the chemical stability of BDP in the concentrated stock solutions (10,000 µg/L). The results (Figure S3A) showed that BDP in water decreased to 22 ± 3% (SD, n=3) of the initial concentration after 24h in a room at 25°C. Thereafter, the rate of decrease slowed markedly to reach 11 ± 12% of the initial BDP concentration at 96h. The pH of the water solution did not significantly affect the degradation rate, which was similar for all the pH values used in the experiment (6, 7.4, and 8.4). BDP solutions prepared in 100% DMF showed minor degradation after 48h, but BDP concentrations remained within ± 15% of the starting value for the duration of the experiment (Figure S3A). Subsequent experiments were performed by increasing the sampling frequency (from 24h to 2-8h) to better characterize the chemical behaviour of BDP in the first 24h (Figure S3B). BDP stability was compared in solutions prepared in 100% water, MeOH:water (10:90, *v:v*) and 100% MeOH at pH 7.4. BDP solutions prepared in 100% MeOH showed minor degradation after 48h, but, as with DMF, BDP concentrations remained within ± 15% of the starting value for the duration of the experiment. However, 10% MeOH did not produce any stabilising effect on BDP concentrations, which had decreased to approximately 50% of the initial BDP concentration after 4h of incubation ($t_{1/2}$=4h), and to 25% after 8h. BDP prepared in water was not detected anymore after 54h of incubation ($t_{1/2}$=4h).

The BDP solutions were also tested for the formation of the metabolites 17-BMP and BOH. 17-BMP was detected at a concentration of <0.2 µg/L after 32h of incubation and at all following sampling times. Thus 17-BMP represented 0.002% of the parent compound BDP in the sample. BOH was never detected at any time point. This result indicates that cleavage of one or both of the propionate groups does not explain the rapid BDP degradation in water and confirms that metabolic activation in the organism is required to efficiently convert BDP into 17-BMP and 17-BMP into BOH. This reaction does not seem to occur abiotically in sterile water (e.g. via hydrolysis), or at least not above negligible rates.

To test the hypothesis that the propionate groups of BDP and 17-BMP increase their susceptibility to degradation, stability experiments were also performed for 17-BMP (Figure S3C) and BOH (Figure S3D). As expected, 17-BMP degraded in 100% water ($t_{1/2}$=40h), but at a slower rate than BDP. After 8h, 17-BMP was present at 87 ± 2% of the starting concentration *versus* 21 ± 4% of BDP. At subsequent sampling times the degradation continued in a linear manner, the concentration reaching 31 ± 1% after 72h. The complete absence of degradation of BOH in both 100% water and MeOH solutions (10% and 100%) (Figure S3D) suggests that the propionate groups increase the susceptibility to degradation.

Pharmacological activity of BDP, 17-BMP and BOH and their transformation products. To investigate if unidentified transformation products of BDP, 17-BMP and BOH retained pharmacological activity, a GR assay was conducted on concentrated solutions prepared in 100% water immediately after preparation and after 96h. The GR activities of the BDP, 17-BMP and BOH solutions after 96h were, respectively, 30.3 ± 9.7%, 53.1 ± 8.6 %, and 91.0 ± 14.9% (mean ± SD; n=3) of the activity quantified on Day 0 (Figure S3E). The concentrations of BDP, BMP and BOH in the same samples after 96h of incubation were 47% ± 4.7%, 60% ± 7.1% and 93% ± 2.8% of the initial concentrations. This result indicates that the pharmacological activities of BDP and 17-BMP decrease simultaneously with the decrease in concentrations of the parent compounds, and that the transformation products of both BDP and 17-BMP do not contribute to the pharmacological activity of the sample to any significant degree.

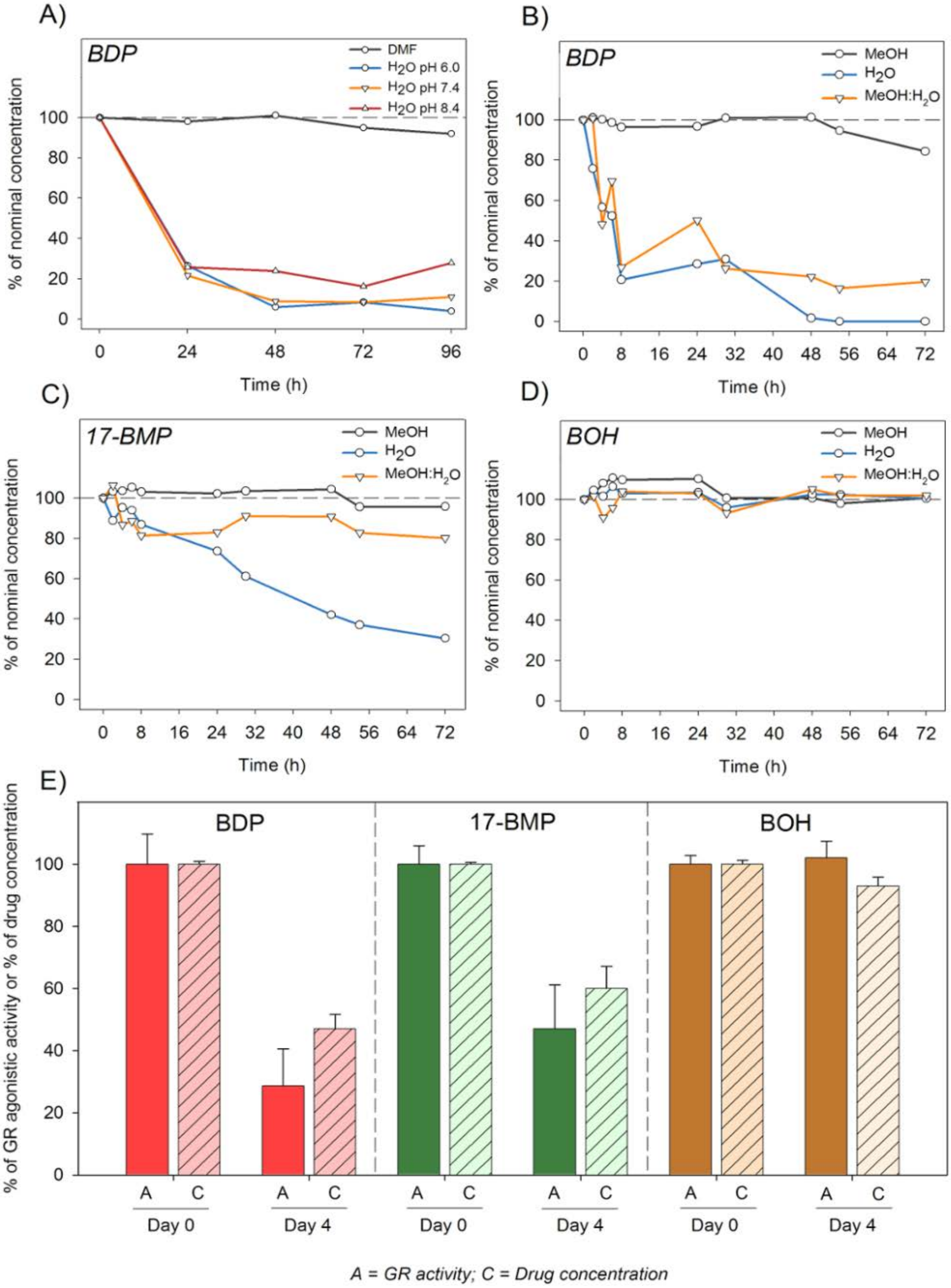

Figure S3. *Stability of BDP (A, B), 17-BMP (C) and BOH (D) in water and solvent-based concentrated solutions over 96h and 72h at 25°C. Panel E illustrates the GR activity and measured concentrations of BDP, 17-BMP and BOH in water solutions immediately after preparation (Day 0) and after 4 days of incubation at 25°C in amber glass bottles. Data are presented as means ± SD (n=3). A indicates the GR activity; C indicates the concentration of the compound in the sample quantified by LC-MS/MS.*

Stability of BDP, 17-BMP and BOH under different storage conditions. Time-dependent degradation of all three compounds was observed when samples were preserved frozen at -20°C (Table S2). Relatively little degradation was observed for samples stored on SPE cartridges after 14 days, with concentrations remaining between 80% and 100% of the initial concentrations. However, significant loss of BDP and 17-BMP had occurred by Day 21 (Table S2). BDP and 17-BMP stored in water showed significant degradation after 4 days and by Day 21 the concentrations had decreased to approximately 50% of the initial value (Table S2). BOH confirmed its higher stability compared to BDP and 17-BMP and degradation was observed only after 21 days in both storage conditions.

Table S2. *Stability of BDP, 17-BMP and BOH (1 µg/L) under different storage conditions.*

Storage type	Time (day)	BDP		17-BMP		BOH	
		%	SD	%	SD	%	SD
SPE (-20°C)	0	100.0	2.4	100.0	1.5	100.0	5.0
	4	95.2	5.9	97.3	3.9	93.8	2.8
	14	93.1	1.7	79.6	2.6	92.4	3.2
	21	57.4	2.0	49.7	2.3	85.4	5.5
Water (-20°C)	0	100.0	2.4	100.0	1.5	100.0	5.0
	4	67.3	3.8	67.1	7.5	90.9	6.1
	14	74.0	4.9	68.5	5.7	92.1	14.9
	21	45.7	5.2	37.4	35.5	74.9	12.5

Quantification of BDP, 17-BMP and BOH in water samples collected from fish tanks.

BDP concentrations in water sample collected during Experiment 1 were approximately 10% of the nominal values for all the three treatment groups (Table S3). In the same water samples, 17-BMP and BOH were also detected at concentrations between 21 and 30 ng/L. At each sampling time, water was collected before the replacement of the stock solutions. This result, combined with those obtained in the stability studies, indicated that, in Experiment 1, fish were exposed to oscillatory concentrations of BDP in water that, in turn, likely produced oscillatory concentrations of BDP, 17-BMP and BOH in fish plasma. In this scenario, fish were exposed to the peak concentrations of BDP (equal to the nominal concentrations) only for 10 hours out of the total 504 hours of exposure. In

Experiment 2, measured water concentrations of BDP were consistently approximately 100% of the nominal concentrations, indicating that fish were exposed to constant concentration of the drug over the 21 days of the experiment. The results obtained in all the described studies were used to estimate the exposure dynamics in both Experiment 1 and Experiment 2 (Figure S3).

Table S3. *Concentrations of BDP in water samples collected during the in vivo exposure studies.*

**Sampling Day**	**EXPERIMENT 1**		**EXPERIMENT 2**	
	**BDP water concentration (ng/L)**		**BDP water concentration (ng/L)**	
	Nominal	*Measured*	*Nominal*	*Measured*
0	0	< LOD	0	< LOD
4	0	< LOD	0	< LOD
7	0	< LOD	0	< LOD
14	0	< LOD	0	< LOD
21	0	< LOD	0	< LOD
0	10	< LOD	10	6.6
4	10	< LOD	10	9.2
7	10	< LOD	10	11.4
14	10	< LOD	10	13.1
21	10	< LOD	10	9.5
0	100	12.1	-	-
4	100	-	-	-
7	100	13.7	-	-
14	100	-	-	-
21	100	9.8	-	-
0	1000	115.9	1000	986.6
4	1000	83.4	1000	942.7
7	1000	101.7	1000	970.7
14	1000	89.9	1000	945.1
21	1000	113.4	1000	815.9

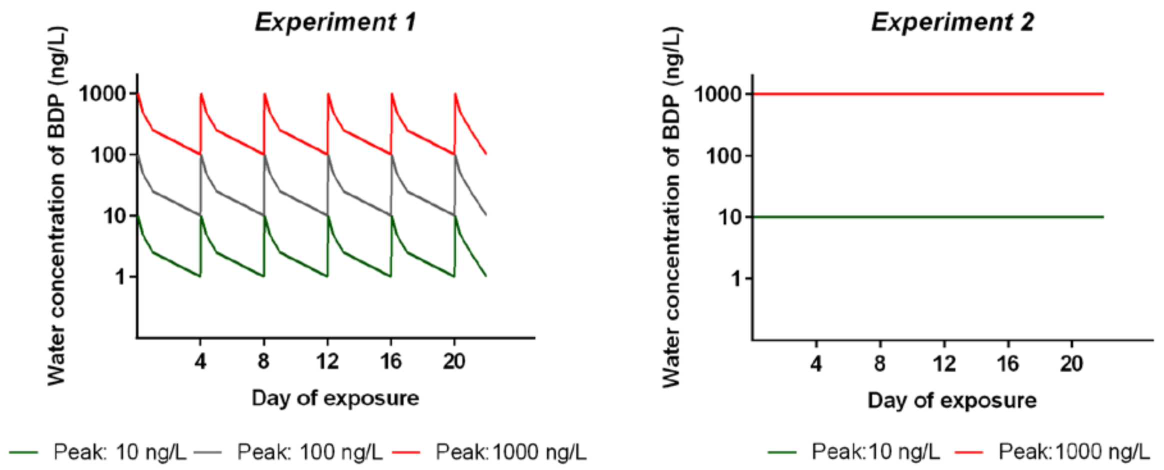

Figure S4. *Water concentrations of BDP in Experiment 1 and Experiment 2.*

BIOLOGY SECTION

Table S4. *Primer sequences used for qPCR reactions.*

**Target gene**	**Forward (5’-3’)**	**Reverse (5’-3’)**	**Reference**
rpl8	CTCCGTCTTCAAAGCCCATGT	TCCTTCACGATCCCCTTGATG	47
GR	GAAAGTCCTTCTGCTCCTGAG	AGTTCTCCTCTCTCTTCACAATG	47
AR	GTGCCATGCGCTTCCAA	CTGACCTTTGTGGGCAAGGA	48
PEPCK	GCTGCTGAACACAAAGGTAAAGTG	GAACCAGTTGACGTGGAAGAT	12
IL6	AGTGCTGAGTTGACATGGGA	TGAGGCAAACCACGACTACA	49
TNFα	CAAGCAATTGGCGAGTGTGT	CAGTTCCACTTTCCTGATTACTCTGA	50

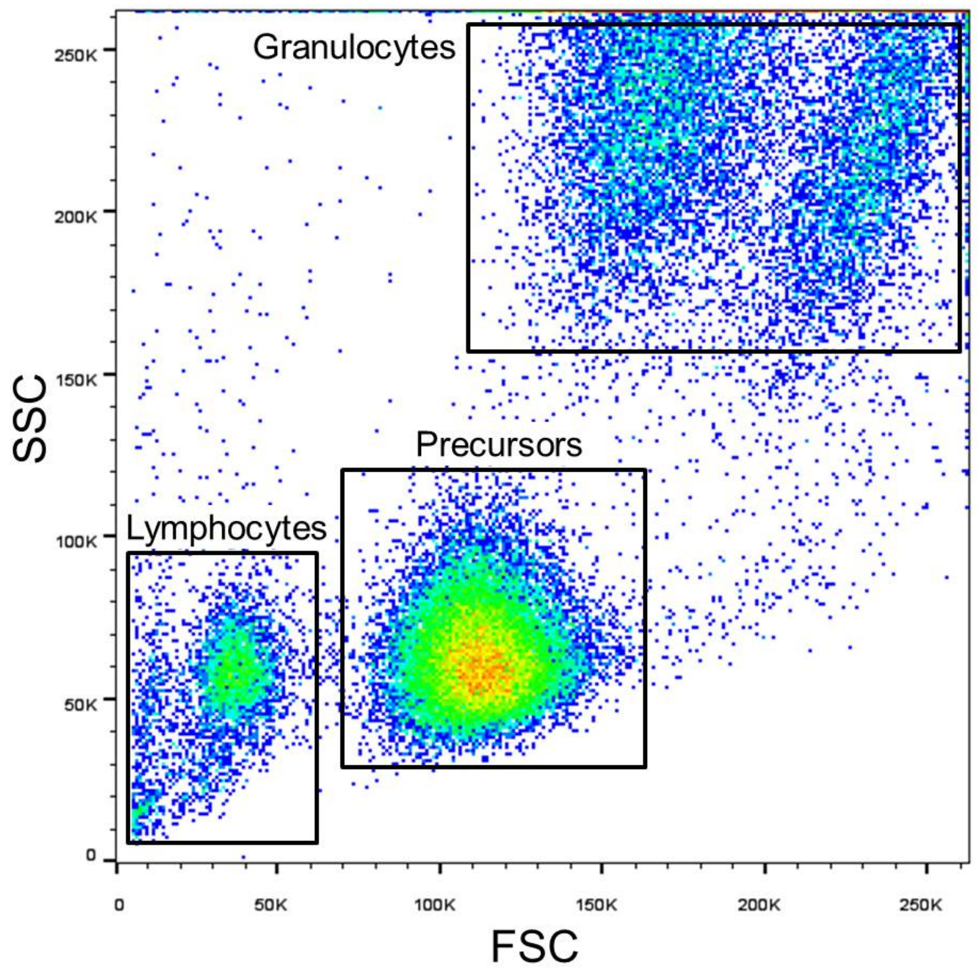

Figure S5. *Forward scatter (FSC) versus side scatter (SSC) profile of fathead minnow white blood cells isolated from blood samples and stained with DiOC6 (3,3'-dihexyloxacarbocyanine iodide). Three main groups of cells were identified and quantified: lymphocytes, white blood cells precursors, and granulocytes.*

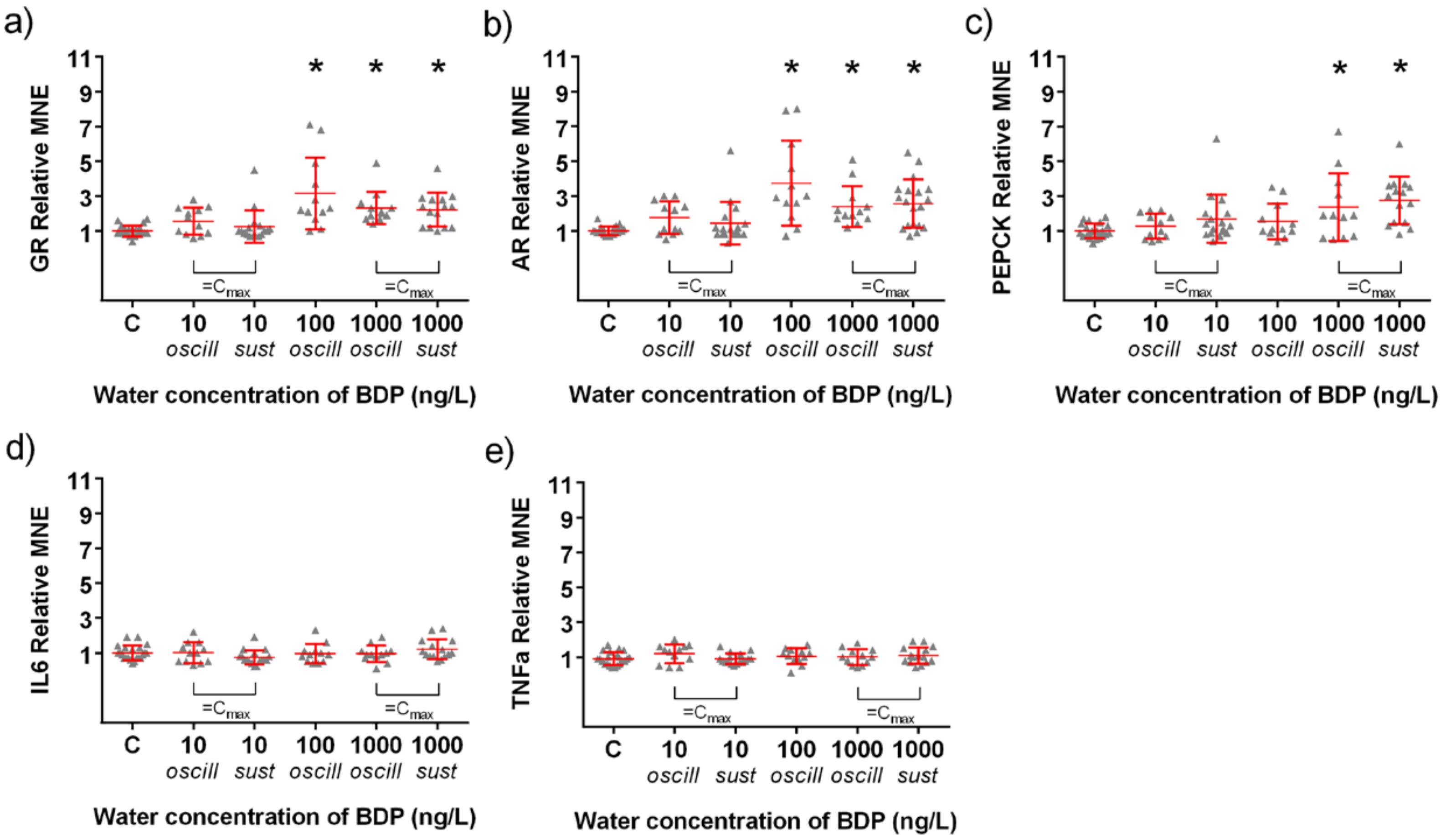

Figure S6. *Relative Mean Normalized Expression (MNE) levels of GR (A), AR (B), PEPCK (C), IL6 (D), and TNFα (E) measured in the liver of fish exposed to increasing plasma concentrations of BDP for 21 days (n=12-20). Triangles indicate individual fish, red lines mean±SD, and asterisks a significant difference versus the control group (p<0.05). The x-axis indicate nominal exposure concentration in water and the exposure dynamic (oscill = oscillatory; sust = sustained).*

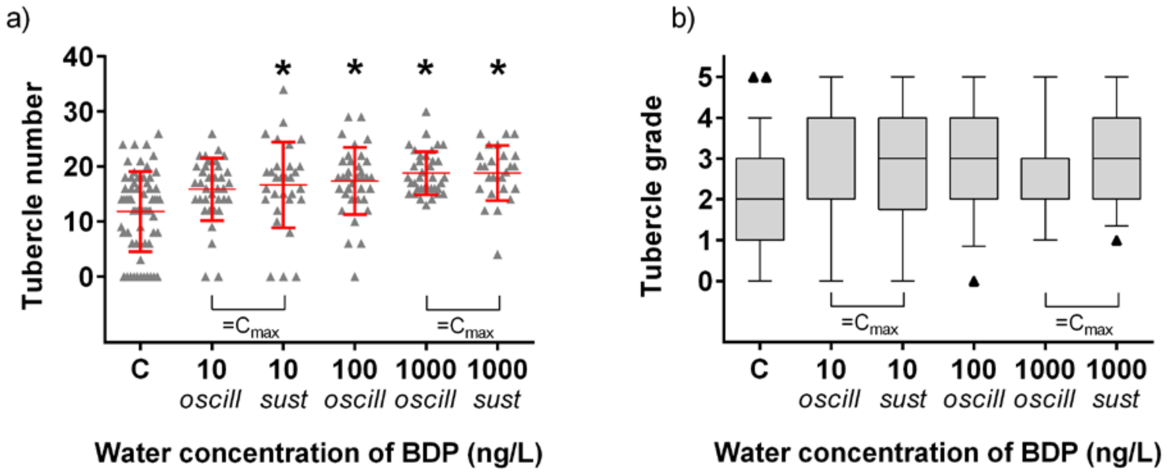

Figure S7. *Tubercle number (A) and tubercle grade (B) measured in male fathead minnows exposed to increasing plasma concentrations of BDP over 21 days (n=36). Triangles indicate individual fish, red lines mean±SD. Boxes represent mean values (full line) with 25th and 75th percentiles. The bars extend to the 10th and 90th percentiles, with outliers represented as black triangles. Asterisks indicate a significant difference versus the control group (p<0.05). The x-axis indicate nominal exposure concentration in water and the exposure dynamic (oscill = oscillatory; sust = sustained).*

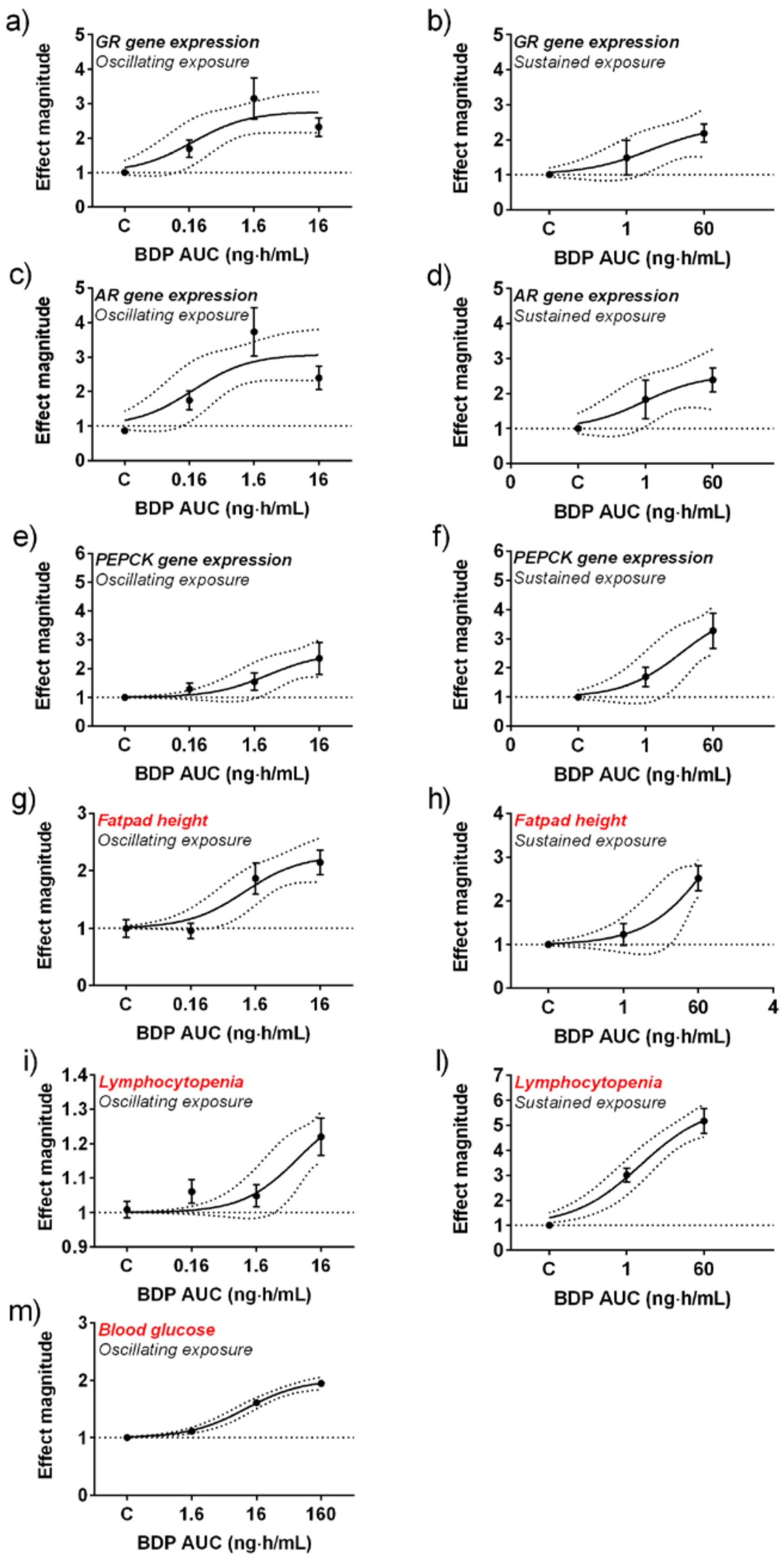

Figure S8. *Concentration response-curve for the endpoints quantified in Experiment 1 (oscillating exposure) and Experiment 2 (sustained exposure). Dots represent mean±SD, solid lines indicate the best-fit regression curve, and the dashed lines the 95% confidence interval. Drug concentrations on the x-axis are expressed as Area Under the Curve (AUC).*

Table S5. *List of effects observed in fish after exposure to the synthetic glucocorticoids beclomethasone dipropionate and dexamethasone.*

**Species**	**Sex**	**Life stage**	**Tissue**	**Endpoint**	**EXTERNAL EXPOSURE**<br>**LOEC (ng/L)**	**INTERNAL EXPOSURE**<br>**Predicted drug C_{max} in fish plasma (ng/mL)**	**INTERNAL EXPOSURE**<br>**Measured drug concentration in fish plasma (ng/mL)**	**Exposure dynamics**	**Exposure duration (days)**
BECLOMETHASONE DIPROPIONATE									
Study reference: present study									
Fathead minnow (*Pimephales promelas*)	M	Adult	Liver	GR (gene expr.)	100	6.5-12.4	-	Oscillatory	21
	M	Adult	Liver	GR (gene expr.)	1,000	6.5-12.4	60±35 (BDP+17-BMP)	Sustained	21
	M	Adult	Liver	AR (gene expr.)	100	6.5-12.4	-	Oscillatory	21
	M	Adult	Liver	AR (gene expr.)	1,000	6.5-12.4	60±35 (BDP+17-BMP)	Sustained	21
	M	Adult	Liver	PEPCK (gene expr.)	1,000	65-124	-	Oscillatory	21
	M	Adult	Liver	PEPCK (gen expr.)	1,000	65-124	60±35 (BDP+17-BMP)	Sustained	21
	M	Adult	Liver	IL6 (gene expr.)	>1,000	>65-124	-	Oscillatory	21
	M	Adult	Liver	IL6 (gene expr.)	>1,000	>65-124	60±35 (BDP+17-BMP)	Sustained	21
	M	Adult	Liver	TNFα (gene expr.)	>1,000	>65-124	-	Oscillatory	21
	M	Adult	Liver	TNFα (gene expr.)	>1,000	>65-124	60±35 (BDP+17-BMP)	Sustained	21
	M	Adult	Liver	HSI	1,000	65-124	-	Oscillatory	21
	M	Adult	Liver	HSI	1,000	65-124	60±35 (BDP+17-BMP)	Sustained	21
	M	Adult	Skin	SSCs (Tubercle number)	100	6.5-12.4	-	Oscillatory	21
	M	Adult	Skin	SSCs (Tubercle number)	10	0.65-1.24	1.2±0.8 (BDP+17-BMP)	Sustained	21
	M	Adult	Skin	SSCs (Tubercle grade)	>1,000	>65-124	-	Oscillatory	21
	M	Adult	Skin	SSCs (Tubercle grade)	1,000	65-124	60±35 (BDP+17-BMP)	Sustained	21
	M	Adult	Skin	SSCs (Fatpad)	100	6.5-12.4	-	Oscillatory	21
	M	Adult	Skin	SSCs (Fatpad)	1,000	65-124	60±35 (BDP+17-BMP)	Sustained	21
	M	Adult	Blood	% of lymphocyte	1,000	65-124	-	Oscillatory	21
	M	Adult	Blood	% of lymphocyte	10	0.65-1.24	1.2±0.8 (BDP+17-BMP)	Sustained	21

Species	Sex	Life stage	Tissue	Endpoint	EXTERNAL EXPOSURE LOEC (ng/L)	INTERNAL EXPOSURE Predicted drug C_{max} in fish plasma (ng/mL)	INTERNAL EXPOSURE Measured drug concentration in fish plasma (ng/mL)	Exposure dynamics	Exposure duration (days)
BECLOMETHASONE DIPROPIONATE									
Study reference: 12, 13									
Fathead minnow (*Pimephales promelas*)	M/F	Adult	Liver	GR (gene expr.)	100	6.5-12.4	-	Oscillatory	21
	M/F	Adult	Liver	PEPCK (gene expr.)	100	6.5-12.4	-	Oscillatory	21
	F	Adult	Liver	Vitellogenin (gene expr.)	100	6.5-12.4	-	Oscillatory	21
	M	Adult	Skin	SSCs (Tubercle number)	10,000	650-1240	-	Oscillatory	21
	M	Adult	Skin	SSCs (Fatpad)	>10,000	>650-1240	-	Oscillatory	21
	F	Adult	Skin	SSCs (Fin spot)	100	6.5-12.4	-	Oscillatory	21
	F	Adult	Skin	SSCs (Ovipositor length)	1,000	65-124	-	Oscillatory	21
	F	Adult	Blood	Vitellogenin	10,000	650-1240	-	Oscillatory	21
	M/F	Adult	Blood	Glucose	1,000	65-124	-	Oscillatory	21
	M/F	Adult	Blood	% of lymphocyte	1,000	65-124	-	Oscillatory	21
	M/F	Adult	Blood	Cortisol	1,000	65-124	-	Oscillatory	21
	F	Adult	-	Egg production	1,000	65-124	-	Oscillatory	21
Study reference: 40									
Rainbow trout	M/F	Juvenile	Liver	PEPCK (gene expr.)	70	5-9	BDP: 0.08±0.02; 17-BMP: 0.20±0.08	Sustained	14
	M/F	Juvenile	Liver	GR (gene expr.)	>650	>42-81	-	Sustained	14
	M/F	Juvenile	Liver	TPP2 (gene expr.)	70	5-9	BDP: 0.08±0.02; 17-BMP: 0.20±0.08	Sustained	14
	M/F	Juvenile	Liver	CD74 (gene expr.)	650	42-81	BDP: 0.4±0.07; 17-BMP: 0.12±0.05	Sustained	14
	M/F	Juvenile	Liver	Glutathione	650	42-81		Sustained	14
	M/F	Juvenile	Liver	Catalase activity	650	42-81		Sustained	14
	M/F	Juvenile	Blood	Glucose	650	42-81		Sustained	14
	M/F	Juvenile	Blood	Hematocrit	>650	>42-81	-	Sustained	14
	M/F	Juvenile	Blood	Hemoglobin	>650	>42-81	-	Sustained	14

Species	Sex	Life stage	Tissue	Endpoint	EXTERNAL EXPOSURE<br>LOEC (ng/L)	INTERNAL EXPOSURE<br>Predicted drug C_{max} in fish plasma (ng/mL)	INTERNAL EXPOSURE<br>Measured drug concentration in fish plasma (ng/mL)	Exposure dynamics	Exposure duration (days)
DEXAMETHASONE									
Study reference: 14									
	M/F	Adult	Reproduction	Egg production	500,000	1528-1662	-	Sustained	21
	M/F	Adult	Reproduction	Fertility	>500,000	>1528-1662	-	Sustained	21
	M/F	Adult	Reproduction	Hatching success	>500,000	>1528-1662	-	Sustained	21
	M/F	Adult	Reproduction	Larvae abnormalities	500,000	1528-1662	-	Sustained	21
	M	Adult	Reproduction	Tubercle score	>500,000	>1528-1662	-	Sustained	21
	M/F	Adult	Reproduction	GSI	500,000	1528-1662	-	Sustained	21
	F	Adult	Reproduction	Plasma E2	500,000	1528-1662	-	Sustained	21
	F	Adult	Reproduction	Ex vivo E2	500,000	1528-1662	-	Sustained	21
	F	Adult	Reproduction	Ex vivo T	>500,000	>1528-1662	-	Sustained	21
	F	Adult	Reproduction	Plasma Vtg	500,000	1528-1662	-	Sustained	21
	M	Adult	Reproduction	Ex vivo T	>500,000	>1528-1662	-	Sustained	21
	M	Adult	Reproduction	Plasma Vtg	>500,000	>1528-1662	-	Sustained	21
	F	Adult	Liver	Vtg	>500,000	>1528-1662	-	Sustained	21
	F	Adult	Liver	ER	>500,000	>1528-1662	-	Sustained	21
	F	Adult	Liver	HMG-CoA	>500,000	>1528-1662	-	Sustained	21
	F	Adult	Gonad	Cyp19a	>500,000	>1528-1662	-	Sustained	21
	F	Adult	Gonad	Vtg	>500,000	>1528-1662	-	Sustained	21
	F	Adult	Liver	GR	50,000<br>(but not at 500,000)	153-166	-	Sustained	21
	M/F	F1 Juvenile	Growth	Growth	500,000	1528-1662	-	Sustained	29
	M/F	F1 adult	Growth	Growth	>500,000	>1528-1662	-	Sustained	29
	M/F	F1 Juvenile	Deformity	Operculum deformity	500,000	1528-1662	-	Sustained	29
	M/F	F1 juvenile	Sex development	Sex ratio	>500,000	>1528-1662	-	Sustained	29

** The value indicates the concentration of BDP+17-BMP in fish plasma sampled on Day 21 of Experiment 1; however, due to the oscillatory nature of the exposure, it cannot be directly correlated with biological effects. AUC values provided in the results are more appropriate for that purpose.*
